# Development and external validation of a risk stratification model for stroke-associated pneumonia

**DOI:** 10.1016/j.isci.2026.116931

**Published:** 2026-07-29

**Authors:** Li Yao, Nan Yang, Naren Ya, Xiao Dong, Wanwan Zhang, Qing Wang, Zhengfei Ma, Yaoming Xu, Yongxin Ma, Mengge Zhang, Fei Gu, Jing Lan, Longfei Wu, Wenbo Zhao, Chuanhui Li, Di Wu, Xunming Ji, Chuanjie Wu

**Affiliations:** 1 Department of Neurology, Xuanwu Hospital, Capital Medical University, Beijing 100053, China; 2 Department of Rehabilitation, Beijing Huimin Hospital, Beijing 100054, China; 3 Department of Neurology, Shihezi People’s Hospital, Shihezi 832000, China; 4 Department of Neurology, Suzhou Municipal Hospital of Anhui Province, Anhui 234000, China; 5 Department of Neurology, Tongliao city People’s Hospital, Tongliao 028000, China; 6 Department of Neurology, Rongcheng County People’s Hospital, Baoding 071700, China; 7 Department of Neurology, Ningjin County People’s Hospital, Xingtai 055550, China; 8 Beijing Institute of Brain Disorders, Capital Medical University, Beijing 100069, China; 9 Department of China-America Institute of Neurology, Xuanwu Hospital, Capital Medical University, Beijing 100053, China; 10 Chinese Academy of Medical Sciences & Peking Union Medical College, Beijing 100730, China

**Keywords:** acute ischemic stroke, stroke-associated pneumonia, prediction model, risk stratification

## Abstract

Stroke-associated pneumonia (SAP) is a common complication after acute stroke and worsens clinical outcomes. We developed and externally validated SENTRY, an early prediction model using routine admission variables and ultra-early chest computed tomography (CT) findings from eight stroke centers. The final model included age, dysphagia, NIHSS (National Institutes of Health Stroke Scale) score, neutrophil-to-lymphocyte ratio, and ultra-early chest CT abnormalities. Compared with a base model, adding chest CT findings improved discrimination (area under the curve [AUC]: 0.811 vs. 0.886) and lowered the Brier score from 0.146 to 0.122. Across validation cohorts, SENTRY showed higher discrimination than established scores (A^2^DS^2^ and ISAN), and decision curve analysis showed greater net benefit across clinically relevant thresholds. SENTRY stratified patients into low-, medium-, and high-risk groups, with stepwise increases in pneumonia incidence. We applied target trial emulation to further illustrate its potential use for risk-enriched trial. A web calculator implements both base and chest CT-inclusive versions of SENTRY to support early risk estimation and risk-stratified workflows.

## Highlights

• Ultra-early chest CT improves SAP prediction beyond admission clinical variables
• SENTRY stratifies patients into clinically distinct SAP risk groups
• SENTRY shows stable discrimination and calibration in external validation
• Risk strata may guide targeted prevention and risk-enriched trial design

## Graphical abstract

**Figure:**
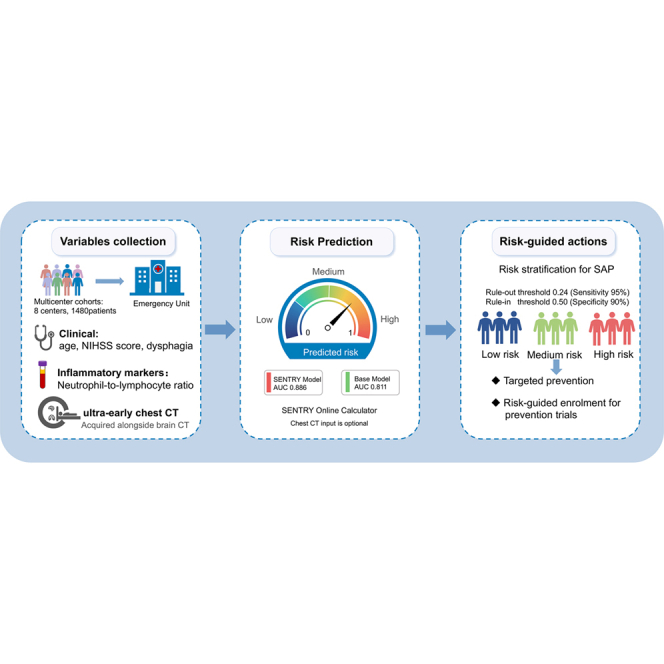

## Teaser

Health sciences; Neurology

## Introduction

Stroke-associated pneumonia (SAP) is a common complication after acute ischemic stroke (AIS), with a pooled global prevalence of 18.3%.^1^ SAP is associated with prolonged hospitalization, increased in-hospital mortality, higher healthcare costs, and poorer functional recovery.^2^^,^^3^^,^^4^ Once SAP occurs, hypoxemia and systemic inflammation may exacerbate secondary brain injury and contribute to neurological deterioration.^5^^,^^6^ As SAP most often occurs within the first 72 h of stroke onset,^7^ timely identification of patients at high risk represents a crucial window for prevention and early management.

Several widely used and independent external validated SAP prediction scores have been proposed, such as Age, Atrial fibrillation, Dysphagia, Sex, and Stroke Severity score (A^2^DS^2^), prestroke Independence, Sex, Age, and National Institutes of Health Stroke Scale (ISAN), Acute Ischaemic Stroke-Associated Pneumonia Score (AIS-APS), and Preventive ANtibacterial THERapy in acute Ischemic Stroke (PANTHERIS).^8^^,^^9^^,^^10^^,^^11^ A^2^DS^2^ and ISAN are pragmatic bedside scores built largely on routinely available admission clinical information reflecting demographics, premorbid status, and stroke severity or functional impairment, including age, sex, atrial fibrillation, dysphagia, National Institutes of Health Stroke Scale (NIHSS) score, and pre-stroke dependency. AIS-APS and PANTHERIS, beyond routinely available bedside variables, also incorporate selected laboratory indices (e.g., admission glucose and white blood cell count, respectively). However, AIS-APS requires multiple input variables and detailed scoring rules, making it relatively complex to calculate and less streamlined for rapid bedside implementation. PANTHERIS was derived from neurological intensive care unit populations, potentially limiting its generalizability to the broader AIS population. Collectively, these established scores remain among the most frequently used and widely cited tools for early SAP risk assessment and are commonly adopted as benchmark comparators.^12^^,^^13^ More recently, several newer SAP prediction models have been developed, including regression and machine learning models.^14^^,^^15^^,^^16^^,^^17^^,^^18^^,^^19^^,^^20^^,^^21^^,^^22^ However, many have limited independent external validation and may be difficult to implement at admission. Some models have been developed in selected populations or specific care settings, which may further limit generalizability.^13^^,^^14^^,^^15^^,^^22^ These gaps highlight the need for tools that can be implemented and generalized across settings.

Beyond neurological severity and functional impairment, AIS is accompanied by early systemic immune alterations, characterized by neutrophilia and lymphopenia, which have been consistently associated with increased susceptibility to infection.^6^^,^^23^^,^^24^ In parallel, several studies have reported pulmonary abnormalities on chest computed tomography (CT) within several hours of stroke onset, including dependent opacities, ground-glass changes, or bronchiolar changes.^25^^,^^26^^,^^27^ Although such findings are not diagnostic of pneumonia, they may reflect ultra-early pulmonary changes, such as dependent atelectasis, mild interstitial edema or congestion, retained secretions, and micro aspiration, which precede the clinical diagnosis of SAP.^28^^,^^29^ Notably, some of these observations were derived from CT angiography, which images only the lung apices and may underestimate early pulmonary involvement.^25^ Despite the potential relevance of these ultra-early immune and pulmonary signals, most SAP prediction models rely on demographic characteristics and neurological severity, without incorporating early markers of infection risk at admission. During the COVID-19 pandemic, routine chest CT was incorporated into the initial evaluation, alongside head CT for patients with AIS at some centers. This unique clinical context provided a natural experiment, enabling systematic ultra-early chest imaging and providing an opportunity to investigate whether early chest CT abnormalities could improve the prediction of SAP.

Randomized trials of prophylactic antibiotics have primarily reported neutral results and do not support routine prophylactic antibiotic use in broad stroke populations.^30^^,^^31^ However, post hoc analyses of the Preventive Antibiotics in Stroke Study (PASS) have suggested that a potential signal may be confined to patients at the highest baseline risk.^32^ These findings highlight the importance of early and accurate risk stratification not only in prioritizing preventive care in clinical practice but also in enabling risk-enriched trial designs and evaluating treatment-effect heterogeneity.

In this context, we aimed to develop and externally validate a multicenter predictive model for SAP and to assess the incremental predictive value of ultra-early chest CT abnormalities beyond conventional bedside variables. Using prespecified risk strata derived from the model, we then applied a target trial emulation framework to examine whether the association between prophylactic antibiotics and SAP differed across strata, informing targeted prevention and future risk-enriched trial designs.

## Results

### Patient demographic and clinical characteristics

**Figure 1:**
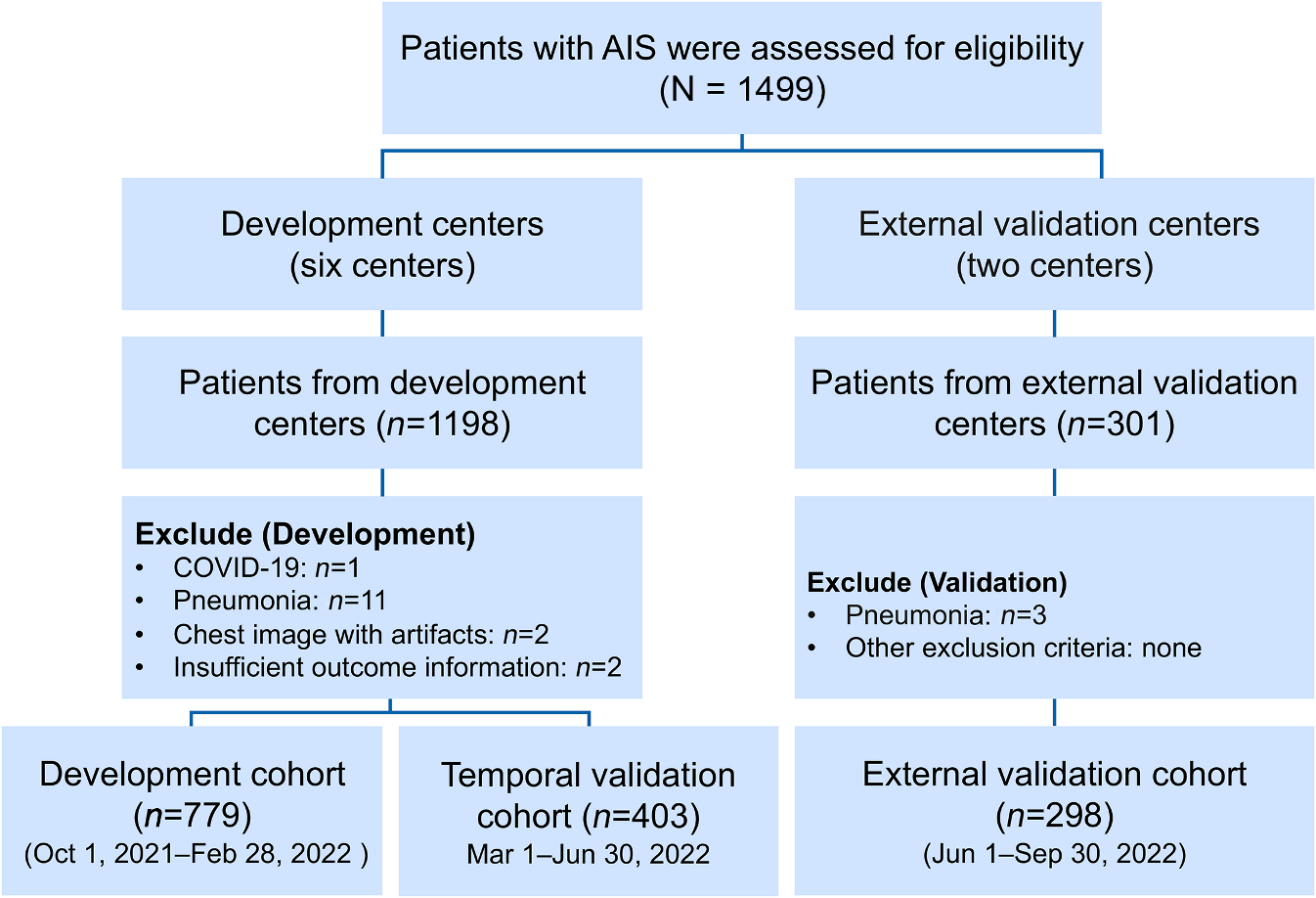
Study flow diagram and cohort derivation “n” denotes the number of patients. AIS, acute ischemic stroke.

Of 1,499 patients screened across eight centers, 1,480 met the inclusion criteria. The final study population comprised a development cohort (*n* = 779), a temporal validation cohort (*n* = 403), and an external validation cohort (*n* = 298) (Figure 1). In the development cohort, the mean age was 66.8 ± 12.6 years; 62.5% of patients were men, and SAP occurred in 28.5% of patients. The median NIHSS score at presentation was 5 (interquatile range [IQR]: 2–11), and dysphagia was present in 20.5% of patients. Chest CT was performed at a median of 6.8 h (IQR: 4.4–10.5) after stroke onset, and 55.5% of patients had early CT abnormalities. In the centralized radiological review, inter-observer agreement for the binary classification of early CT abnormalities was 95.6%, with a Cohen’s κ of 0.912. Baseline characteristics in the temporal and external validation cohorts were broadly comparable to those in the development cohort, although the NIHSS score was slightly higher and the proportion of participants with dysarthria was lower in the temporal and external validation cohorts (Table 1).

**Table 1:** Characteristics of participating patients in our study

Variables	Development cohort (*n* = 779)	Temporal validation cohort (*n* = 403)	External validation cohort (*n* = 298)
**Demographics**			

Age (years), mean ± SD	66.8 ± 12.6	65.4 ± 12.4	64.6 ± 12.1
Male, *n* (%)	487 (62.5%)	265 (65.7%)	197 (66.1%)

**Baseline function, *n* (%)**			

Pre-stroke mRS (0), *n* (%)	615 (78.9%)	313 (77.7%)	244 (81.9%)
Pre-stroke mRS (1–2), *n* (%)	120 (15.4%)	62 (15.4%)	43 (14.4%)
Pre-stroke mRS (≥3), *n* (%)	44 (5.7%)	28 (6.9%)	11 (3.7%)

**Stroke characteristics**			

Time from stroke onset to admission, hours, median (IQR)	5.1 (2.8–8.1)	5.6 (2.7–9.0)	5.3 (2.3–9.6)
NIHSS score median (IQR)	5 (2–11)	8 (4–18)	7 (3–15)
Dysphagia, *n* (%)	160 (20.5%)	56 (13.8%)	49(16.4%)

**Stroke location**			

Anterior, *n* (%)	607 (77.9%)	321 (79.7%)	229 (76.8%)
Posterior, *n* (%)	172 (22.1%)	82 (20.3%)	69 (23.2%)

**Laboratory indices (10**^**9**^**/L)**			

Neutrophil count, median (IQR)	5.7 (4.2–6.1)	4.7 (3.3–5.7)	4.1 (3.0–5.3)
Lymphocyte count, median (IQR)	1.4 (1.0–1.8)	1.1 (0.9–1.5)	1.0 (0.9–1.2)

**Imaging finding**s			

Time from stroke onset to chest CT scan, hours, median (IQR)	6.8 (4.4–10.5)	7.5 (5.1–10.9)	6.4 (3.8–10.5)
Early abnormalities, *n* (%)	432 (55.5%)	221 (54.8%)	160 (53.7%)

**Medical comorbidities**			

Current smoking, *n* (%)	278 (35.7%)	122 (30.3%)	113 (37.9%)
Diabetes, *n* (%)	227 (29.1%)	101 (25.1%)	88 (29.5%)
Hypertension, *n* (%)	518 (66.5%)	276 (68.5%)	194 (65.1%)
Atrial fibrillation, *n* (%)	88 (11.3%)	38 (9.4%)	24 (8.1%)
COPD, *n* (%)	21 (2.7%)	6 (1.5%)	6 (2.0%)
History of stroke, *n* (%)	205 (26.3%)	79(19.6%)	71(23.8%)

**Outcomes**			

SAP, *n* (%)	222 (28.5%)	101 (25.1%)	73 (24.5%)

Abbreviations: COPD, chronic obstructive pulmonary disease; mRS, modified Rankin scale; SAP, stroke-associated pneumonia.

### Model development and selected predictors

Neutrophil-to-lymphocyte ratio (NLR) was markedly right skewed (Figure S1A) and was, therefore, log-transformed. Restricted cubic spline (RCS) indicated a non-linear association for log-transformed NLR (*p* = 0.044), whereas age and NIHSS score showed approximately linear associations with SAP. C-reactive protein and procalcitonin were excluded due to >30% missingness; no imputation was required for the remaining predictors. White blood cell count was excluded from the Least Absolute Shrinkage and Selection Operator (LASSO) analysis due to collinearity with NLR. Using 10-fold cross-validated LASSO with the λ.1se criterion, five predictors, namely age, NIHSS score, dysphagia, log-transformed NLR, and early chest CT abnormalities, were selected for inclusion in the final multivariable logistic regression model (Figure S2). The associations (including non-linear effects) between each predictor and SAP in the final SENTRY model fitted in the full development cohort are shown in Figures S1B–S1F.

### Model performance and validation

**Figure 2:**
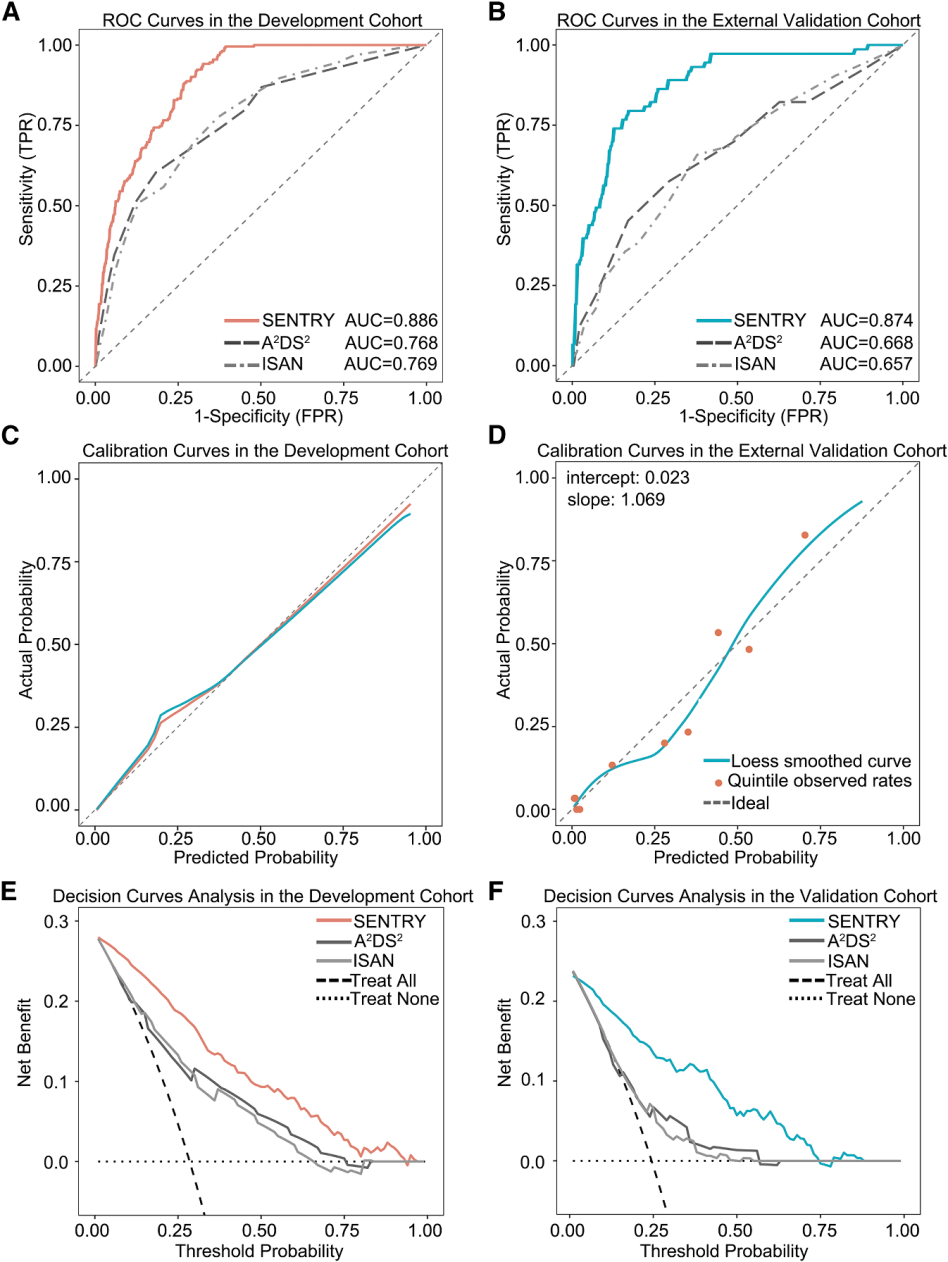
Discrimination, calibration, and decision curve analysis of the SENTRY model (A and B) Receiver operating characteristic (ROC) curves comparing SENTRY with A^2^DS^2^ and ISAN in the development cohort (A) and external validation cohort (B). FPR, false positive rate; TPR, true positive rate; AUC, area under the ROC curve. (C and D) Calibration plots of the SENTRY model, showing apparent and bootstrap bias-corrected calibration compared with the ideal line in the development cohort (C), and loess-smoothed calibration with quintile-based observed event rates in the external validation cohort (D). (E and F) Decision curve analysis demonstrating the net benefit of SENTRY, A^2^DS^2^, and ISAN across a range of threshold probabilities in the development cohort (E) and validation cohort (F).

In the development cohort, the SENTRY model demonstrated good discrimination, with an area under the curve (AUC) of 0.886 (95% CI, 0.864–0.909), and good overall performance, with a Brier score of 0.122 (95% CI, 0.110–0.135) (Figure 2A). Calibration was acceptable, as shown in the calibration plot (Figure 2C). Internal validation using 1,000 bootstrap resamples showed minimal overfitting, with an optimism-corrected AUC of 0.881.

To assess model transportability across centers, we performed leave-one-center-out internal-external cross-validation within the development cohort. Model performance was consistent across the six held-out centers, with a mean AUC of 0.877 ± 0.016 (mean ± SD) and a mean Brier score of 0.128 ± 0.010 (mean ± SD). Center-specific discrimination and calibration results are presented in Figure S3 and Table S1.

In the temporal validation cohort, SENTRY achieved an AUC of 0.862 (95% CI, 0.821–0.903) (Figure S4A) and a Brier score of 0.125 (95% CI, 0.109–0.143). Calibration remained stable, with a calibration intercept of −0.030 and a slope of 0.940 (Figure S4B).

In the external cohort, SENTRY achieved an AUC of 0.874 (95% CI, 0.829–0.920) (Figure 2B) and a Brier score of 0.119 (95% CI, 0.103–0.142). Calibration was well maintained, with an intercept of 0.023 and a slope of 1.069 (Figure 2D).

### Incremental value of ultra-early chest CT abnormalities

Compared with the CT-free SENTRY model (base model including age, NIHSS score, dysphagia, and log-transformed NLR), the inclusion of ultra-early chest CT abnormalities was associated with higher discrimination and improved overall accuracy across cohorts. In the development cohort, the AUC increased from 0.811 to 0.886 (ΔAUC = 0.075), while the Brier score decreased from 0.146 to 0.122. Similar patterns were observed in the temporal validation cohort (AUC: from 0.725 to 0.862; Brier score: from 0.167 to 0.125) and the external validation cohort (AUC: from 0.734 to 0.874; Brier score: from 0.163 to 0.119) (Figures S5A–S5C). Decision curve analysis indicated a higher net benefit for the SENTRY model than for the base model across clinically relevant risk thresholds (Figures S5D–S5F).

### Comparison with established bedside scores

A^2^DS^2^ and ISAN are widely used bedside scores and can be computed using routinely available variables, facilitating consistent implementation across centers. We, therefore, selected A^2^DS^2^ and ISAN as primary benchmark comparators. In the external validation cohort, SENTRY demonstrated higher discrimination than A^2^DS^2^ and ISAN (AUCs: 0.668 [95% CI, 0.593–0.744] and 0.657 [95% CI, 0.584–0.730], respectively; *p* < 0.001 for both comparisons, DeLong’s test) (Figure 2B; Table S5). Corresponding comparisons in the development and temporal validation cohorts are shown in Figures 2A and S4A. Decision curve analysis indicated higher net benefit for SENTRY than A^2^DS^2^ and ISAN across clinically relevant threshold probabilities in the validation cohorts (Figures 2E, 2F, and S4C).

When chest CT was unavailable, we further compared the CT-free SENTRY base model against A^2^DS^2^ and ISAN. The base model showed stronger discrimination in the temporal validation cohort (AUC: 0.725 vs. A^2^DS^2^ [AUC = 0.665] and ISAN [AUC = 0.647]) and the external validation cohort (AUC: 0.734 vs. A^2^DS^2^ [AUC = 0.668] and ISAN [AUC = 0.657]) (Figure S6; Table S6). Differences in AUCs were assessed using DeLong’s test (Table S6). Across clinically relevant threshold probabilities, the base model provided greater net benefit than A^2^DS^2^ and ISAN in decision curve analyses (Figures S6D–S6F).

### Two-threshold strategy for accurate risk stratification of SAP

Using the prespecified two-threshold framework, patients were categorized into low-, medium-, and high-risk groups based on predicted scores from the SENTRY model. The lower threshold was defined to achieve 95% sensitivity (rule-out threshold), and the upper threshold was defined to achieve 90% specificity (rule-in threshold). Risk strata were applied unchanged across cohorts.

**Figure 3:**
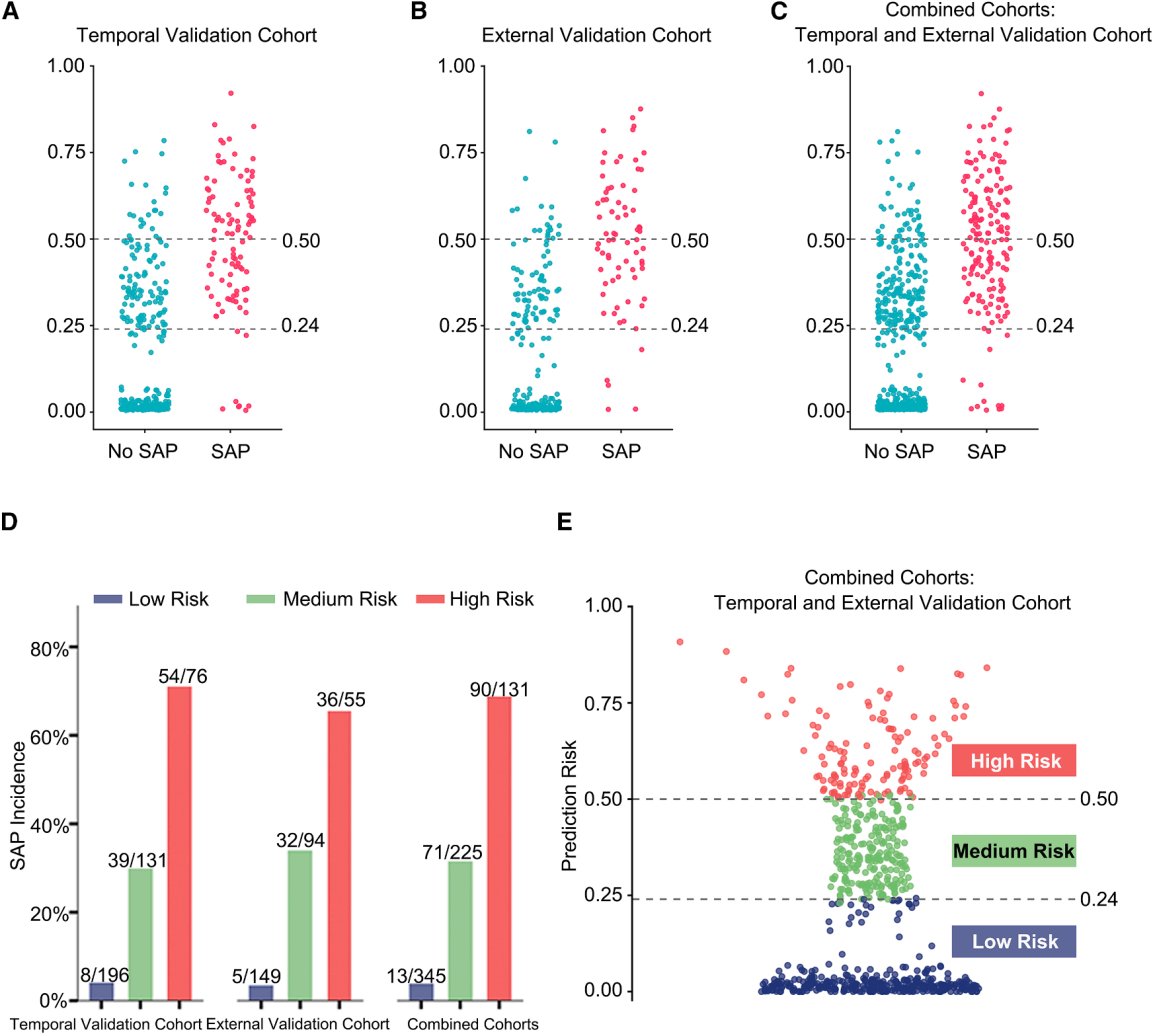
Risk stratification of patients using a prespecified two-threshold strategy (A–C) Distribution of predicted risks among patients with and without stroke-associated pneumonia (SAP) in the temporal validation cohort, external validation cohort, and the combined cohorts. Dashed horizontal lines indicate the prespecified rule-out (0.24) and rule-in (0.50) thresholds. (D) Incidence of SAP across low-, medium-, and high-risk groups in the temporal and external validation cohorts. Bars represent the proportion of patients who developed SAP in each risk stratum, with numbers above the bars indicating SAP events/total patients within each risk stratum. (E) Distribution of predicted risks in the combined validation cohorts, illustrating separation of patients into low-, medium-, and high-risk groups according to the two-threshold framework.

In the temporal validation cohort, the observed incidence of SAP increased stepwise across risk strata, from 4.1% (8/196) in the low-risk group to 29.8% (39/131) in the medium-risk group and 71.1% (54/76) in the high-risk group (Figures 3A and 3B). A similar gradient was observed in the external validation cohort, with SAP occurring in 3.4% (5/149), 34% (32/94), and 65.5% (36/55) of patients in the low, medium, and high-risk groups, respectively (Figure 3D).

When the temporal and external validation cohorts were combined, predicted risk values spanned a wide range and differed between patients with and without SAP (Figure 3C), with low-, medium-, and high-risk strata corresponding to distinct intervals of predicted probability (Figure 3E).

### Web-based implementation of SENTRY

To facilitate clinical implementation, we developed a web-based SENTRY calculator that provides both CT-free and CT-inclusive prediction pathways. The calculator reports the predicted probability of SAP and classifies patients into low-, medium-, or high-risk groups according to the prespecified rule-out and rule-in thresholds. The online SENTRY calculator is available at https://sentry.shinyapps.io/SENTRY/. A screenshot of the calculator interface is provided in Figure S10.

### Target trial emulation

Among 701 eligible patients, 101 (14.4%) initiated prophylactic antibiotics within 24 h, and 600 (85.6%) did not. In the high-risk stratum (predicted risk ≥ 0.50), 53 of 131 patients initiated antibiotics within 24 h, compared with 48 of 570 in the low- and medium-risk strata (predicted risk < 0.50). Unweighted SAP incidence was higher in the high-risk stratum than in the low- and medium-risk strata (68.7% [90/131] vs. 14.7% [84/570]).

In inverse probability of treatment weighted (IPTW) analyses, prophylactic antibiotic initiation was associated with a lower odds of SAP in the high-risk stratum (OR: 0.403, 95% CI: 0.186–0.859; *p* = 0.020), whereas no association was observed in the non-high-risk stratum (OR: 1.003, 95%: CI 0.375–2.284; *p* = 0.994). Consistent findings were observed in propensity score matched (PSM) analyses: in the high-risk stratum, SAP occurred in 26/48 (56.5%) of treated patients versus 35/46 (76.1%) of untreated patients (OR: 0.409, 95% CI: 0.163–0.984; *p* = 0.050), whereas no risk reduction was observed in the low- and medium-risk groups (treated: 10/47 [21.3%] vs. untreated: 9/47 [19.2%]; OR: 1.141, 95% CI: 0.414–3.181; *p* = 0.797). Covariate balance after weighting and matching is shown in Figures S7 and S8 (standardized mean differences before and after adjustment).

### Sensitivity analyses

Sensitivity analyses yielded similar performance estimates across alternative modeling specifications. Using the λ_min penalty produced an AUC of 0.890 (95% CI, 0.868–0.912) and a Brier score of 0.120 (95% CI, 0.107–0.133). When the model was refit without RCS, the AUC was 0.883 (95% CI, 0.860–0.905) and the Brier score was 0.124 (95% CI, 0.112–0.137). Receiver operating characteristic (ROC) curves and calibration curves are shown in Figure S9.

## Discussion

In this multicenter study, we developed and externally validated SENTRY, an early prediction model for SAP that integrates routinely admission variables with ultra-early chest CT abnormalities. Model performance was consistent across centers and remained stable in temporal and external validation. Using prespecified thresholds, we classified patients into low-, medium-, and high-risk strata based on predicted probabilities. To support clinical use, we prespecified two probability thresholds to categorize patients into low-, medium-, and high-risk strata for early triage and prioritization of preventive supportive care. This framework also provides a basis for risk-enriched evaluation of preventive strategies.

Clinical factors such as age, stroke severity, and dysphagia reflect neurological impairment and aspiration risk, which are well-established determinants of post-stroke infection.^8^^,^^9^^,^^10^^,^^33^^,^^34^ In addition to these clinical vulnerabilities, AIS triggers a cascade of systemic and pulmonary changes that create a permissive environment for early pneumonia.^35^ Stroke induces rapid immune suppression, mediated through the activation of sympathetic pathways and the hypothalamic-pituitary-adrenal axis,^36^ which can lead to early lymphopenia, impaired innate immune function, and increased susceptibility to infection in the acute phase.^36^^,^^37^ These early immune changes provide a biological rationale for incorporating NLR as a readily available marker of acute immune dysregulation that complements clinical vulnerability.

Stroke may also exert early effects on the pulmonary structure and physiology. Experimental studies have described early pulmonary edema, inflammatory infiltration, and disruption of alveolar homeostasis shortly after cerebral ischaemia,^38^^,^^39^ and similar non-pneumonic abnormalities have been observed on early chest CT in patients with AIS.^25^^,^^26^^,^^27^ Dependent atelectasis, peribronchial thickening, focal opacities, and consolidation are frequently observed soon after symptom onset, and the presence of such findings has been associated with a higher subsequent risk of SAP.^25^^,^^26^^,^^27^ Collectively, this evidence provides a mechanistic basis for considering early chest CT abnormalities as complementary prognostic information at presentation.

In our cohorts, benchmarking against the commonly used bedside SAP risk scores A^2^DS^2^ and ISAN suggested that demographics and neurological severity captured a substantial proportion of risk (Table S7). Yet, residual variation remains among patients with similar age and stroke severity.^27^^,^^40^ Our findings indicate that early inflammatory status, such as NLR, and ultra-early chest CT abnormalities detectable soon after stroke, may provide complementary information that is not explicitly represented in traditional bedside tools. Notably, the chest CT signal in our study was coded as a simple binary indicator based on routine clinical interpretation and did not require any specialized image post-processing. Additionally, several SAP prediction models have been reported in recent years. However, many studies have been derived from single-center datasets or restricted to specific care pathways or patient subgroups, such as AIS patients undergoing thrombectomy, AIS patients with type 2 diabetes mellitus, or those with atrial fibrillation (Table S8).^13^^,^^14^^,^^15^^,^^22^^,^^41^ These limitations, together with limited independent external validation and variable reporting of calibration and clinical utility across clinically relevant decision thresholds, may constrain transportability and bedside use. Admission time implementation may also be challenging when predictors are not uniformly available at baseline or are influenced by post-admission management, such as nasogastric tube feeding and mechanical ventilation.^19^^,^^42^ Against this backdrop, the SENTRY model integrates admission available inflammatory markers and an ultra-early chest CT signal, addressing practical barriers to implementation while maintaining performance in independent cohorts.

Evidence from prophylactic trials remains inconclusive, and trial design features may have limited the ability to detect benefit in truly high-risk patients. The PASS trial enrolled a broad acute stroke population with generally mild stroke severity (median NIHSS score: 5 [IQR: 3–9]) and randomized patients within 24 h of symptom onset to preventive ceftriaxone or standard care.^30^ The Prophylactic antibiotics after acute stroke for reducing pneumonia in patients with dysphagia (STROKE-INF) trial restricted enrollment to dysphagic patients and started antibiotics within 48 h. The relatively mild stroke severity in PASS may have reduced the overall pneumonia event rate, whereas the later treatment window in STROKE-INF may have missed the optimal window for early prevention. These design features may therefore have attenuated a potential treantment benefit confined to a well-defined high-risk subgoup.^32^ Therefore, a better early risk model could help identify patients at the highest risk of SAP and provide a framework for exploring whether preventive strategies might have different effects across the risk groups.

We further translated individual predicted probabilities into clinically interpretable risk strata by using a prespecified two-threshold framework. In both the temporal and external validation cohorts, SAP incidence increased monotonically from low- to high-risk strata. Stratum-specific event rates were broadly comparable between cohorts, supporting preserved risk gradients and transportable calibration. Notably, the framework identified a substantially low-risk subgroup with a low observed event rate and a high-risk subgroup that accounted for a large proportion of subsequent SAP events, facilitating pragmatic prioritization in early stroke care. Consistent with this, decision curve analyses suggested that threshold-based use of the model could yield net benefit across clinically relevant probability ranges, supporting its potential utility for early triage, rather than risk estimation alone. In practice, such stratification can inform differentiated preventive intensity: low-risk patients may be managed with standard monitoring and routine care, whereas high-risk patients may warrant closer surveillance and enhanced supportive prevention, including timely swallowing assessment, aspiration-prevention and airway-care bundles, oral hygiene, early mobilization and rehabilitation, and optimization of nutrition and positioning. Additional pharmacologic prophylaxis could be evaluated in risk-enriched randomized trials to establish benefits and harms.

Importantly, this risk stratification not only continues risk estimation but also provides actionable categories at the point of care. In the acute stroke setting, where time and resources are constrained, translating predicted probabilities into discrete risk strata may facilitate rapid decision-making and prioritization. The identification of a large low-risk subgroup with a very low observed SAP incidence supports a rule-out function, potentially reducing unnecessary preventive interventions and antibiotic exposure. Conversely, the high-risk stratum concentrated a substantial proportion of subsequent SAP events, providing a clinically and methodologically suitable population for intensified preventive strategies. This marked risk gradient underpins the feasibility of risk-enriched trial designs, in which intervention effects may be more readily detectable than in unselected stroke populations.

To support implementation across heterogeneous care settings, we developed SENTRY as a web-based calculator with two complementary configurations. A CT-free base model, relying on bedside variables and an inflammatory marker, is intended for patients for whom a chest CT is unavailable, and an imaging-augmented model for patients for whom a chest CT is obtainable. In our validation cohorts, the CT-free model showed higher discrimination than commonly used bedside scores (A^2^DS^2^ and ISAN), supporting a streamlined approach that remains practical for early use. The calculator reports predicted risk along with the rule-out and rule-in thresholds to enable rapid, reproducible risk categorization at the point of care. This can be readily integrated into digital decision-support workflows and facilitate transparent, auditable triage. Importantly, the tool is intended to support early risk stratification and prioritization of preventive supportive care; additionally, it may serve as a pragmatic foundation for risk-enriched randomized trial planning of adjunct pharmacologic prophylaxis.

In summary, we developed and validated the SENTRY model by integrating clinical variables and ultra-early chest CT abnormalities to enable early risk stratification for SAP. The model demonstrated good performance in temporal and external validation cohorts, which supports its potential to identify patients at high risk who may benefit from intensified monitoring and targeted preventive strategies; a low-risk subgroup with a very low event rate may avoid unnecessary preventive interventions and their associated treatment burden. Moreover, SENTRY may support risk-enriched design of future prevention-focused randomized trials. Prospective evaluation is warranted to assess its impact on clinical decision-making and patient outcomes.

### Limitations of the study

This study has several limitations. First, ultra-early chest CT was systematically available at our centers during the COVID-19 period due to admission infection-control policies; however, routine chest CT is not part of acute stroke workflows in many settings, and the availability and timing of imaging may differ elsewhere. To enhance applicability, we, therefore, provide a CT-free model based on bedside variables and NLR when chest CT is unavailable, framing chest CT as an optional, opportunistically available enhancement, rather than a required component. In addition, although the binary CT classification was adopted to enhance inter-reader consistency and multicenter feasibility, it may have obscured differences among specific radiologic phenotypes; future prospective studies should collect CT abnormality subtypes in a structured manner. Second, although the eight participating centers covered different regions of China and included both tertiary and regional hospitals, all cohorts were derived from a single national healthcare system and were predominantly composed of Han Chinese patients. Validation in other countries and care pathways is needed to further establish transportability. Third, this study was retrospective and relied on routinely collected clinical data, which may have introduced selection bias and incomplete documentation. Prospective validation in a real-world, real-time data-capture setting is, therefore, warranted to confirm the predictive performance, clinical utility, and safety of the SENTRY model before broader implementation. Accordingly, we are currently designing a prospective validation study to further evaluate its performance under routine clinical workflow.

## Resource availability

### Lead contact

For further information or resource-related inquiries, please contact the lead contact, Prof. Chuanjie Wu (wuchuanjie@ccmu.edu.cn).

### Materials availability

No novel or unique reagents were generated in this study.

### Data and code availability

• Individual-level clinical data in this study are not publicly available due to patient privacy restrictions, institutional data governance policies, and ethics requirements. De-identified data may be made available from the corresponding author upon reasonable request and subject to IRB and institutional approval.
• All original code has been deposited at GitHub (https://github.com/cmu-sentry/sap) and is publicly available.
• Any additional information required to reanalyze the data reported in this paper is available from the lead contact upon request.

## Acknowledgments

We thank all participating patients and the clinical staff at the collaborating centers for their contributions to data collection and study coordination. We acknowledge funding from the Beijing Natural Science Foundation (JQ24041), Xuanwu Hospital Talent Convergence Program (HZ2025ZCBJ002), Noncommunicable Chronic Diseases- National Science and Technology Major Project (2030ZD0505400-2023ZD0505403), and the science and technology plan project in Inner Mongolia (2025YFSH0081).

## Author contributions

L. Yao, N. Yang, X. Ji, and C. Wu have fully reviewed and verified the study data and are responsible for the integrity and accuracy of the data analysis; X. Ji and C. Wu contributed to the study’s concept and design; X. Dong, W. Zhang, Z. Ma, Y. Xu, Y. Ma, M. Zhang, N. Ya, and F. Gu performed data collection; X. Dong, Q. Wang, W. Zhang, L.Wu, W. Zhao, and D. Wu participated in the analysis and interpretation of the data; L.Yao. was responsible for drafting of the manuscript, and L. Wu, W. Zhao, J. Lan, D. Wu, X. Ji, and C. Wu provided critical revisions for important intellectual content; L. Yao, N. Yang, and X. Dong performed the statistical analysis; C. Wu acquired funding for the study. All authors have modified and approved the final manuscript and accept responsibility for the decision to submit for publication.

## Declaration of interests

The authors declare no competing interests.

## STAR★Methods

### Key resources table

**Table:** 

REAGENT or RESOURCE	SOURCE	IDENTIFIER
**Other**		

EHR data	Eight participating centers: Xuanwu Hospital; Shihezi Municipal Hospital; Rongcheng County People’s Hospital; The Second Hospital of Hebei Medical University; Ningjin County People’s Hospital; Suzhou Municipal Hospital of Anhui Province; Central Hospital of Xiangtan; First Affiliated Hospital of Soochow University	Access upon IRB approval

**Deposited data**		

Original analysis code	This study	GitHub: http://github.com/cmu-sentry/sap

**Software and algorithms**		

R	R Foundation for Statistical Computing	R 4.4.2
ggplot2	https://cran.r-project.org/web/packages/ggplot2/index.html	NA
pROC	https://web.expasy.org/pROC/	NA
rms	https://cran.r-project.org/web/packages/rms/index.html	NA
pmsampsize	https://CRAN.R-project.org/package=pmsampsize	NA
Shiny	https://shiny.posit.co/	NA

### Experimental model and study participant details

#### Study design and ethics

We conducted a retrospective, multicenter cohort study across eight tertiary stroke centers in China (Table S2). Adults with AIS consecutively admitted to six centers between Oct 1, 2021, and Feb 28, 2022, constituted the development cohort, and those between Mar 1 and May 31, 2022, were assigned to the temporal validation cohort. Patients with AIS admitted to two additional centers between June 1 and Sep 30, 2022, were included in the external validation cohort. This temporal–geographic validation design allowed assessment of model performance across different time periods and centers, thereby evaluating robustness to population shifts. Center-specific study periods were defined by local COVID-19 screening policies that mandated admission chest CT for emergency stroke presentations. Outside these policy windows, chest CT was not routinely required.

The study protocol was approved by the ethics committee of Xuanwu Hospital ([2021]053) and by the institutional review boards of all participating centers; informed consent was waived because anonymized retrospective data were used. The study was registered at ClinicalTrials.gov (NCT07321275) and reported in accordance with TRIPOD+AI guidelines.^43^

#### Participants

Patients aged 18 years and older with neuroimaging-confirmed AIS, who underwent chest CT within 24 hours of onset, were included. For patients with more than one admission during the study period, only the first eligible admission was included. Patients were excluded for: (1) pneumonia, supported by compatible clinical features (e.g., fever/leukocytosis, purulent sputum, respiratory symptoms) and radiographic findings considered consistent with pneumonia on imaging; (2) unavailable or incomplete chest CT images; or (3) insufficient information to ascertain SAP within 7 days after stroke onset.

### Method details

#### Chest CT acquisition and evaluation

Chest CT scans were obtained at all eight participating centers using multidetector CT scanners. They were commonly acquired alongside brain CT scans on admission. During the COVID-19 pandemic, chest CT was incorporated into the initial emergency imaging work-up at these centers. Examinations were obtained without intravenous contrast, typically during an inspiratory breath-hold to minimize motion artefacts. Scanning coverage extended from the thoracic inlet to the costophrenic angles. Images were reconstructed as standard axial sections with 5-mm slice thickness for routine review, with additional thin-section reconstructions (0.625–1.000 mm) for lung parenchymal assessment.

#### Ultra-early chest CT assessment

All baseline chest CT images from the participating centers were collected and assessed through a centralized, independent, blinded radiological review process. Baseline chest CT images were independently reviewed by two radiologists with more than 10 years of experience in chest imaging, who were blinded to clinical information and study outcomes. Before image review, the two radiologists received standardized training and underwent a calibration session using representative cases to harmonize the CT-positive and CT-negative definitions and the review approach. Classification of abnormalities was based solely on imaging appearance. Discrepancies were resolved by consensus, and when agreement could not be reached, a third senior reviewer adjudicated.

CT findings were dichotomized as positive or negative according to prespecified criteria. A scan was considered positive if any acute pulmonary abnormality was present, including dependent opacities, atelectatic change, ground-glass opacity, focal or patchy consolidative change, bronchiolar abnormalities, pleural effusion, or visible airway secretions or mucus plugging. Findings considered non-acute or chronic on radiologic assessment, such as emphysema, fibrotic change, linear scarring, pleural thickening, or calcified granulomas, were classified as negative unless accompanied by an acute abnormality.

Patients with clinically evident pneumonia at baseline were excluded according to prespecified clinical criteria. Accordingly, baseline chest CT abnormalities were treated as ultra-early pulmonary imaging signals potentially relevant to subsequent SAP risk, rather than as a radiologic diagnosis of pneumonia. A binary classification approach was adopted to enhance inter-reader consistency and improve robustness across centers. Inter-observer agreement was assessed using the initial independent CT-positive/CT-negative classifications before consensus adjudication.

#### Candidate predictors, sample size, and data handling

Clinical data were extracted from electronic medical records and standardized case report forms using a uniform template across centers. Candidate predictors were identified through a comprehensive literature review and availability across both cohorts, and then refined through a Delphi process involving neurologists, radiologists, respiratory physicians, intensivists, and infectious disease specialists. Predictors were obtained from the initial emergency assessment. Full details of predictor variable handling and candidate predictors are provided in Table S3.

An *a priori* sample size calculation was performed.^44^ We assumed an event rate of 18.3% SAP, based on a recent systematic review and meta-analysis; an anticipated C-statistic of 0.80^45^; and a maximum of 15 candidate predictor parameters. Based on these inputs, the minimum sample size for new model development was estimated to be 709 patients, corresponding to 130 events.

Candidate predictors with more than 30% missing data were prespecified for exclusion. Missing outcome data were not imputed. Non-linearity was assessed by likelihood ratio tests comparing spline and linear specifications. Restricted cubic splines (RCS) with two internal knots were used when *p* < 0.05; otherwise, predictors were entered as linear terms.

#### Study outcomes

The primary outcome was stroke-associated pneumonia (SAP), defined as a new pneumonia diagnosed within 7 days after stroke onset, defined according to the modified Centers for Disease Control and Prevention (CDC) criteria (Table S4).^28^^,^^29^ SAP was diagnosed when all of the following were present: (1) systemic signs of infection; (2) new or worsening respiratory manifestations; and (3) new radiographic pulmonary abnormalities on chest imaging. Because all participants underwent baseline chest CT scans, radiographic evidence was primarily derived from them.

### Quantification and statistical analysis

All statistical analyses were performed using R version 4.4.2. Unless otherwise specified, n represents the number of patients. Continuous variables are summarized as mean ± SD or median (IQR), as appropriate, with mean or median representing the measure of center and SD or IQR representing dispersion. Categorical variables are summarized as n (%). Model discrimination was assessed using the area under the receiver operating characteristic curve (AUC), calibration using calibration plots, calibration intercepts and slopes, and overall performance using the Brier score. AUCs were compared using DeLong’s test. Associations in the target trial emulation were estimated using inverse probability of treatment weighting (IPTW)-weighted logistic regression models with robust standard errors and reported as odds ratios (ORs) with 95% confidence intervals. All statistical tests were two-sided, and *p* < 0.05 was considered statistically significant. Statistical details, including exact *n* values, statistical tests, measures of center, dispersion, and precision, are provided in the Results, Tables, Figures, and figure legends.

#### Model development and validation

We developed a multivariable logistic regression model to predict the risk of SAP using easily available admission variables. To derive a concise and clinically applicable model while reducing the risk of overfitting, we used LASSO-penalized regression to select a subset of predictors; predictors with nonzero coefficients were retained and refitted in a standard multivariable logistic regression model to derive the final coefficients and predicted risks.

Model performance in the development cohort was assessed by discrimination, quantified using the area under the receiver operating characteristic curve (AUC), and by overall accuracy, measured using the Brier score. Model calibration was evaluated using calibration plots. Internal validation was performed by bootstrap resampling (1000 iterations) to obtain optimism-corrected estimates of model performance.

To evaluate transportability across centers within the development dataset, we applied an internal–external cross-validation strategy using a leave-one-center-out approach. In each iteration, the model was trained on data from all centers except one and tested in the excluded center. This procedure was repeated until each center had served as the validation set once. Model performance was assessed in each held-out center, with center-specific discrimination and calibration evaluated using the AUC and calibration plots, including calibration intercepts and slopes.

The final model was subsequently evaluated in an independent temporal validation cohort and an external validation cohort using identical predictor and outcome definitions. In each validation cohort, model discrimination, calibration, and overall accuracy were assessed using the same performance metrics as in the development cohort, with uncertainty quantified by bootstrap resampling to derive 95% confidence intervals. Calibration was evaluated by plotting observed versus predicted proportions of stroke-associated pneumonia across ten risk groups, with smoothed calibration curves generated using locally estimated scatterplot smoothing (LOESS). We performed decision curve analysis (DCA) to compare the net benefit of the SENTRY model with that of the comparator model across a range of risk thresholds.

Clinical risk scores used for comparison included the A^2^DS^2^ and ISAN scores, according to their original published criteria.^9^^,^^10^ Discrimination was assessed using the AUC with 95% confidence intervals, and AUCs were compared using DeLong’s test.

To support clinical implementation, we developed a web-based risk calculator that provides two complementary prediction pathways. A clinically based model uses routinely available admission variables, while a CT-enhanced model integrates early chest CT findings to maximize predictive performance when imaging data are available. Both models are implemented within the same application to accommodate variability in real-world data availability.

#### Two-threshold risk categorization

To enhance clinical usability, we applied a prespecified two-threshold framework to the SENTRY predicted scores, classifying patients into low-, medium-, and high-risk groups. The lower threshold was selected in the development cohort to achieve 95% sensitivity and was used as a rule-out threshold to identify patients at sufficiently low predicted risk. The upper threshold was selected in the development cohort to achieve 90% specificity and was used as a rule-in threshold to identify patients at high predicted risk. Patients with predicted risks between the lower and upper thresholds were classified as medium risk. The resulting thresholds were then fixed and applied unchanged to the temporal and external validation cohorts. The risk categories were used to describe event rates across strata and to support risk-enriched trial analyses.

#### Target trial emulation

We conducted a target trial emulation to demonstrate how model-based risk stratification could inform the design of a future risk-enriched randomized trial of prophylactic antibiotics. Time zero (T0) was defined as the time of completion of the chest CT scan. Predictor values were ascertained at or before T0. Eligible participants were adults with acute ischemic stroke who had no diagnosis of SAP before or at T0.

Two strategies were emulated using a prespecified 24-hour decision window after T0: initiation of prophylactic antibiotics within 24 hours after T0 and no initiation of prophylactic antibiotics within 24 hours after T0. Antibiotic initiation after the 24-hour window was not considered prophylactic for the purposes of defining these strategies; participants who developed SAP within 24 hours after T0 were excluded to align eligibility with the decision window. Using the prespecified risk categories, we conducted stratified analyses to illustrate how enrichment based on predicted risk might affect trial feasibility and precision.

As treatment assignment was not randomized, confounding was addressed using IPTW based on a propensity score estimated from baseline covariates measured at T0. Weighted regression models with robust standard errors were used to estimate the association between strategy initiation within 24 hours and subsequent SAP, overall and within the prespecified risk strata. Follow-up started at 24 hours after T0 and continued until 7 days after stroke onset.

#### Sensitivity analyses

To assess the robustness of our findings, we prespecified the following sensitivity analyses: (1) refitting the prediction model using a simplified functional form for continuous predictors by removing restricted cubic splines (RCS) (e.g., modeling age without spline terms while retaining log-transformed NLR); (2) repeating penalized variable selection with the penalty parameter selected at λ_min; and (3) re-estimating the target trial emulation effects using 1:1 propensity score matching instead of inverse probability of treatment weighting, using the same baseline covariates as in the primary analysis.

### Additional resources

The study was registered at ClinicalTrials.gov under identifier NCT07321275: https://clinicaltrials.gov/study/NCT07321275. The SENTRY web calculator is available at: https://sentry.shinyapps.io/SENTRY/.
