# Supplementary material for: Development and external validation of a risk stratification model for stroke-associated pneumonia
Source: iScience. 2026 Jul 29;29(8):116931. doi: 10.1016/j.isci.2026.116931 (PMC13451795; doi:10.1016/j.isci.2026.116931)
Supplement: Supplementary file 1 [file mmc1.pdf]

iScience, Volume *29*

Supplemental information

Development and external validation of a risk stratification model for stroke-associated pneumonia

Li Yao, Nan Yang, Naren Ya, Xiao Dong, Wanwan Zhang, Qing Wang, Zhengfei Ma, Yaoming Xu, Yongxin Ma, Mengge Zhang, Fei Gu, Jing Lan, Longfei Wu, Wenbo Zhao, Chuanhui Li, Di Wu, Xunming Ji, and Chuanjie Wu

Figure S1: Multivariable associations between selected predictors and outcome in the SENTRY model

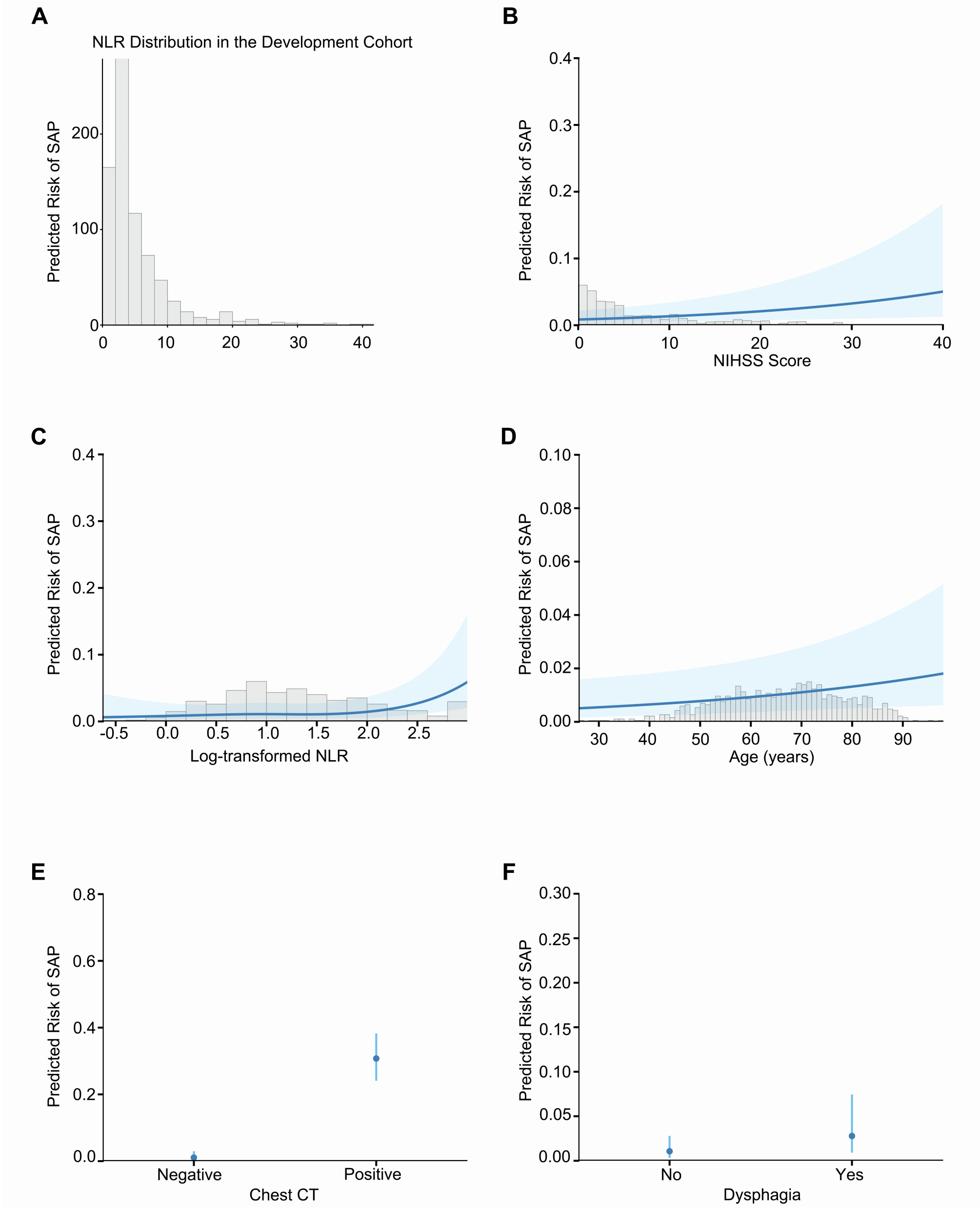

A: Distribution of neutrophil-to-lymphocyte ratio in the development cohort. NLR, Neutrophil-to-lymphocyte ratios.

B-D: Adjusted associations of continuous predictors: NIHSS score (B), log-transformed NLR (C), and age (D), with the predicted risk of SAP. Solid lines represent model-estimated risks, with shaded areas indicating 95% confidence intervals; overlaid histograms show the distribution of each predictor in the development cohort. NIHSS: National Institutes of Health Stroke Scale,

E-F: Predicted risks of SAP according to binary predictors: ultra-early chest CT abnormalities (E) and dysphagia (F), shown as point estimates with 95% confidence intervals.

Figure S2: LASSO model cross-validation curve and coefficient profiles

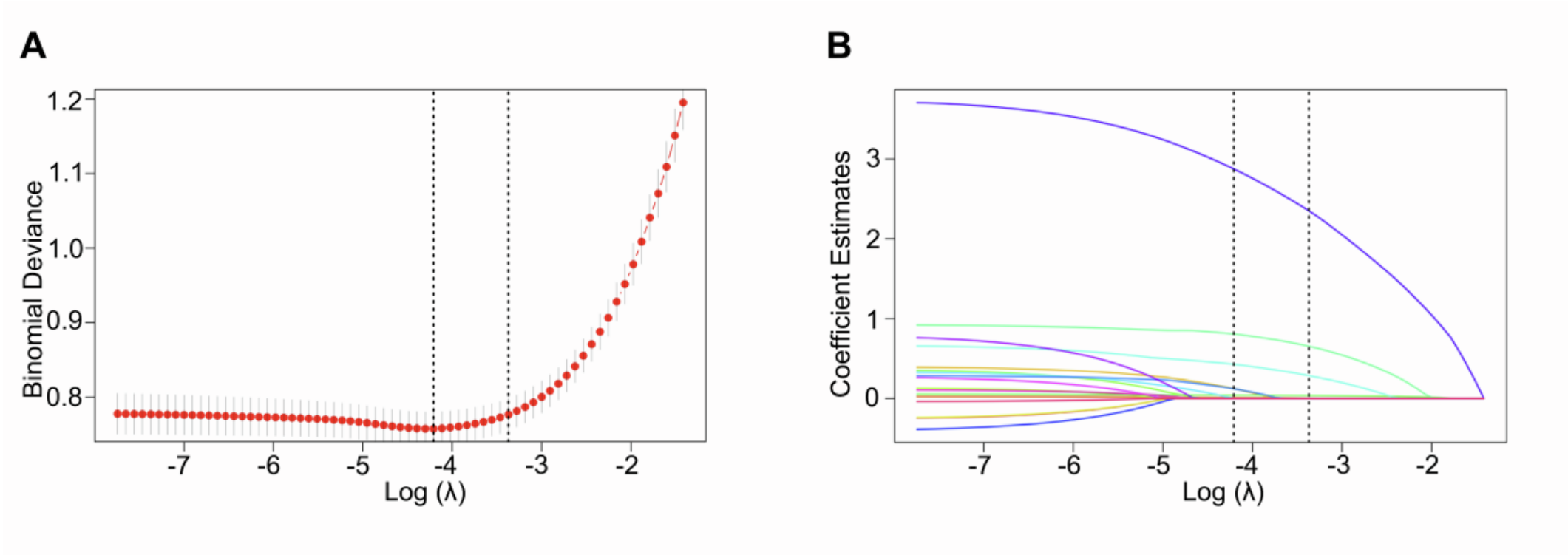

A: Cross-validation curve showing mean binomial deviance across log(λ). Vertical dashed lines denote λ. min (minimum deviance) and λ.1se (the most regularised model within 1 standard error).

B: Coefficient profiles across log(λ), illustrating coefficient shrinkage as λ increases.

Figure S3: ROC curves for each center from leave-one-center-out internal–external validation

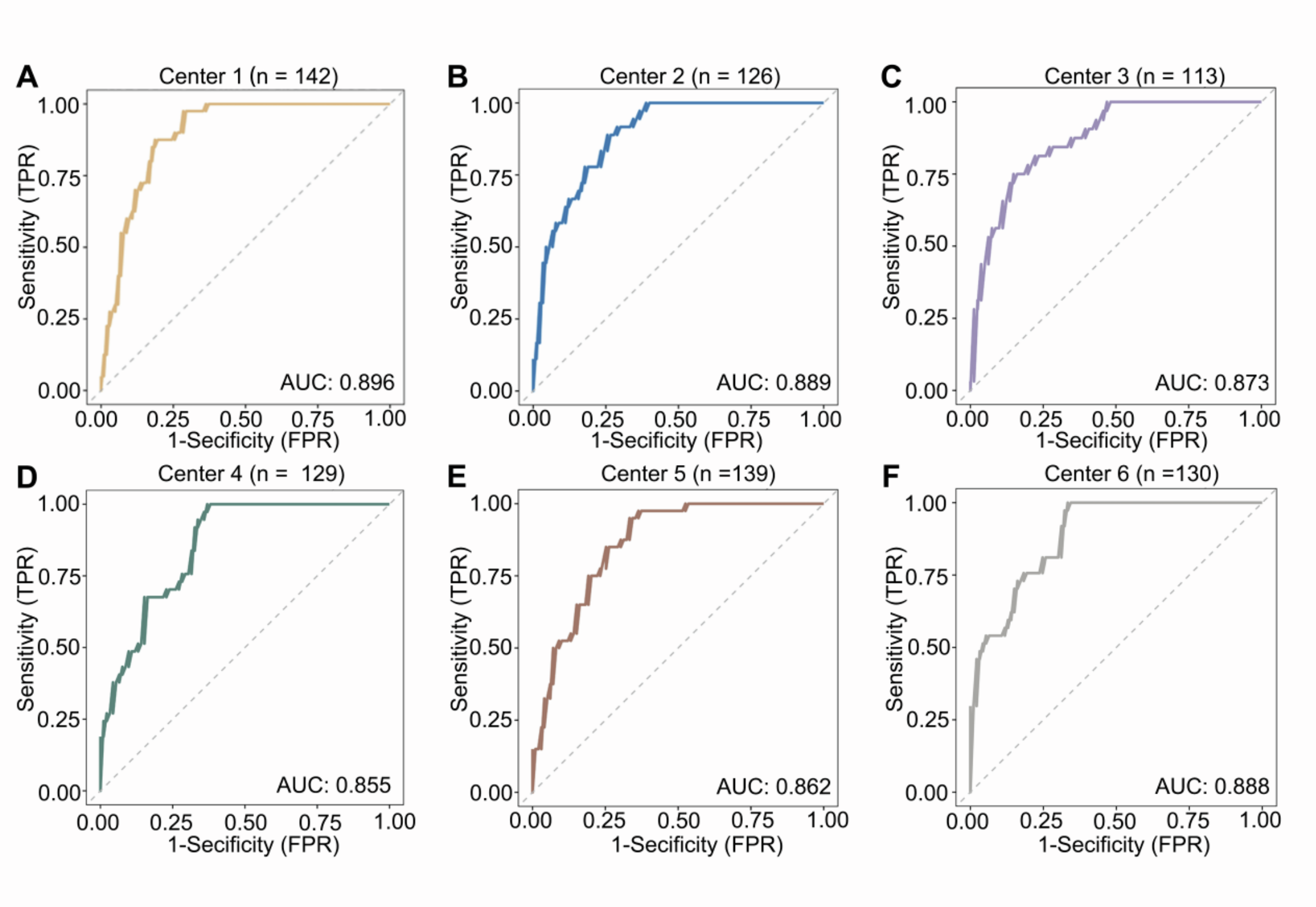

A–F: Receiver operating characteristic (ROC) curves for each participating center, generated by refitting the model using data from the remaining centers and evaluating performance in the held-out center. Panels A–F correspond to Centers 1–6, respectively, with sample sizes indicated in each panel. Each plot displays the ROC curve for the held-out center and the corresponding area under the curve (AUC). The diagonal dashed line indicates the no-discrimination line. Centers 1 to 6 correspond to the following institutions: Xuanwu Hospital, Shihezi Municipal Hospital, Rongcheng County People's Hospital, The Second Hospital of Hebei Medical University, Ningjin County People's Hospital, and Suzhou Municipal Hospital.

Abbreviations: FPR, false positive rate; TPR, true positive rate; n, number of patients.

Figure S4: Performance of the SENTRY Model in the Temporal Validation Cohort

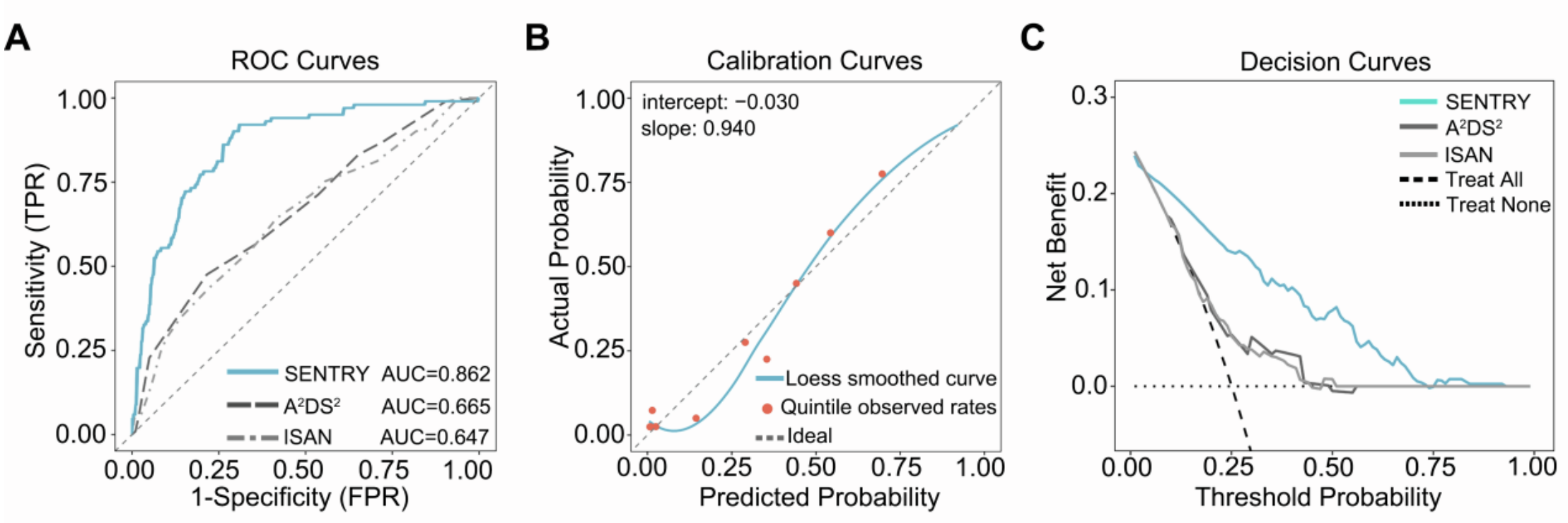

A: Receiver operating characteristic (ROC) curves comparing the discrimination of SENTRY with the A²DS² and ISAN scores.

B: Calibration plot of the SENTRY model, showing the agreement between predicted and observed probabilities. The solid line represents the locally weighted scatterplot smoothing (LOESS) curve, points denote observed event rates across quintiles of predicted risk, and the dashed line indicates ideal calibration.

C: Decision curve analysis comparing the net benefit of SENTRY with A²DS² and ISAN across a range of threshold probabilities. The dashed and dotted lines represent the “treat-all” and “treat-none” strategies, respectively.

Figure S5: Incremental value of ultra-early chest CT abnormalities across cohorts

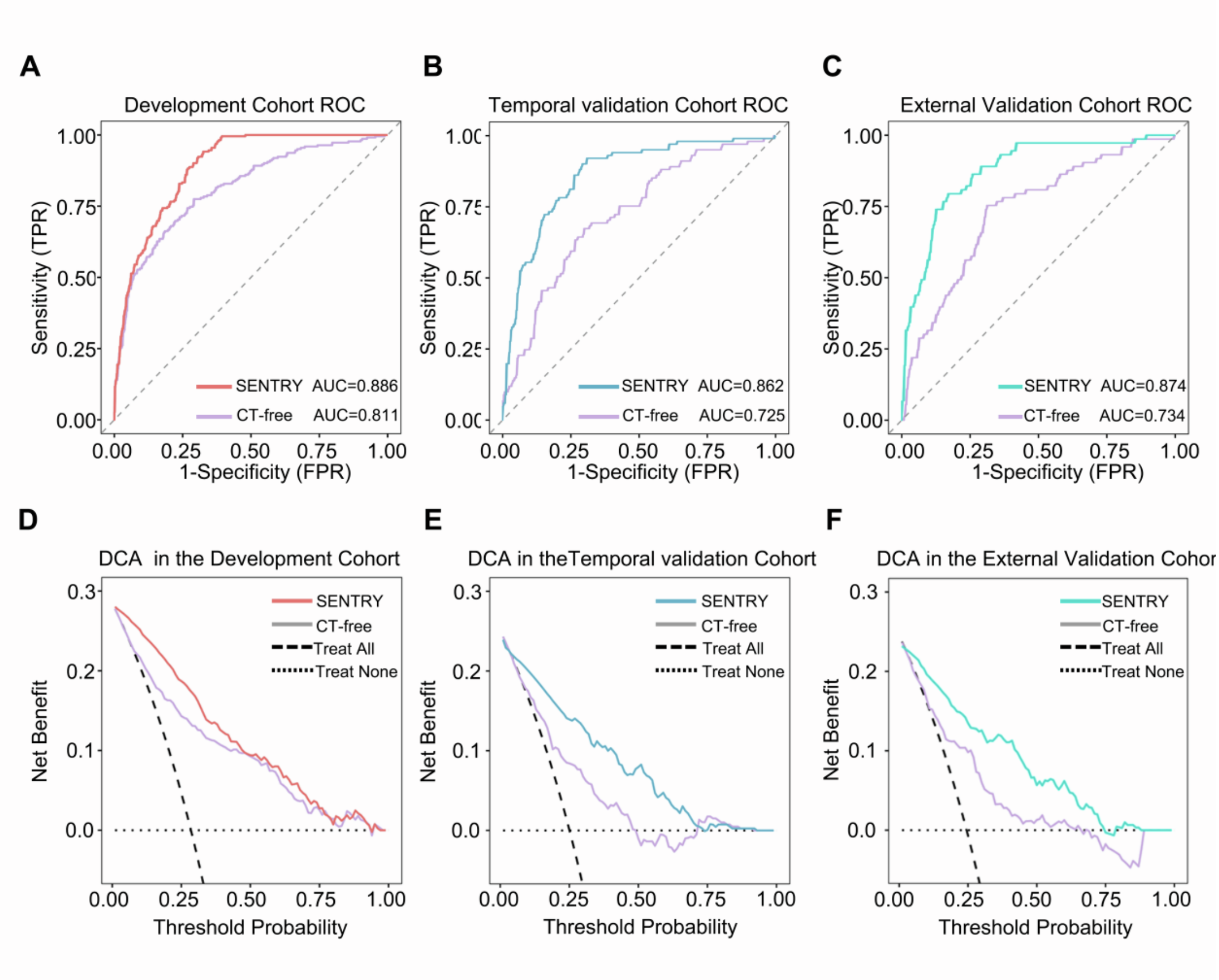

A–C: Receiver operating characteristic (ROC) curves comparing the discrimination of the SENTRY model with a CT-free base model in the development cohort (A), temporal validation cohort (B), and external validation cohort (C). Area under the curve (AUC) values are shown in the ROC panels.

D–F: Decision curve analyses (DCA) comparing the net benefit of the two models across the development cohort (D), temporal validation cohort (E), and external validation cohort (F).

The base model included age, National Institutes of Health Stroke Scale (NIHSS) score, dysphagia, and the log-transformed neutrophil-to-lymphocyte ratio (NLR); the SENTRY model additionally incorporated ultra-early chest CT abnormalities. In the DCA panels, dashed and dotted lines represent the “treat-all” and “treat-none” strategies, respectively.

Figure S6: Discrimination and clinical utility of the CT-free SENTRY base model compared with A²DS² and ISAN.

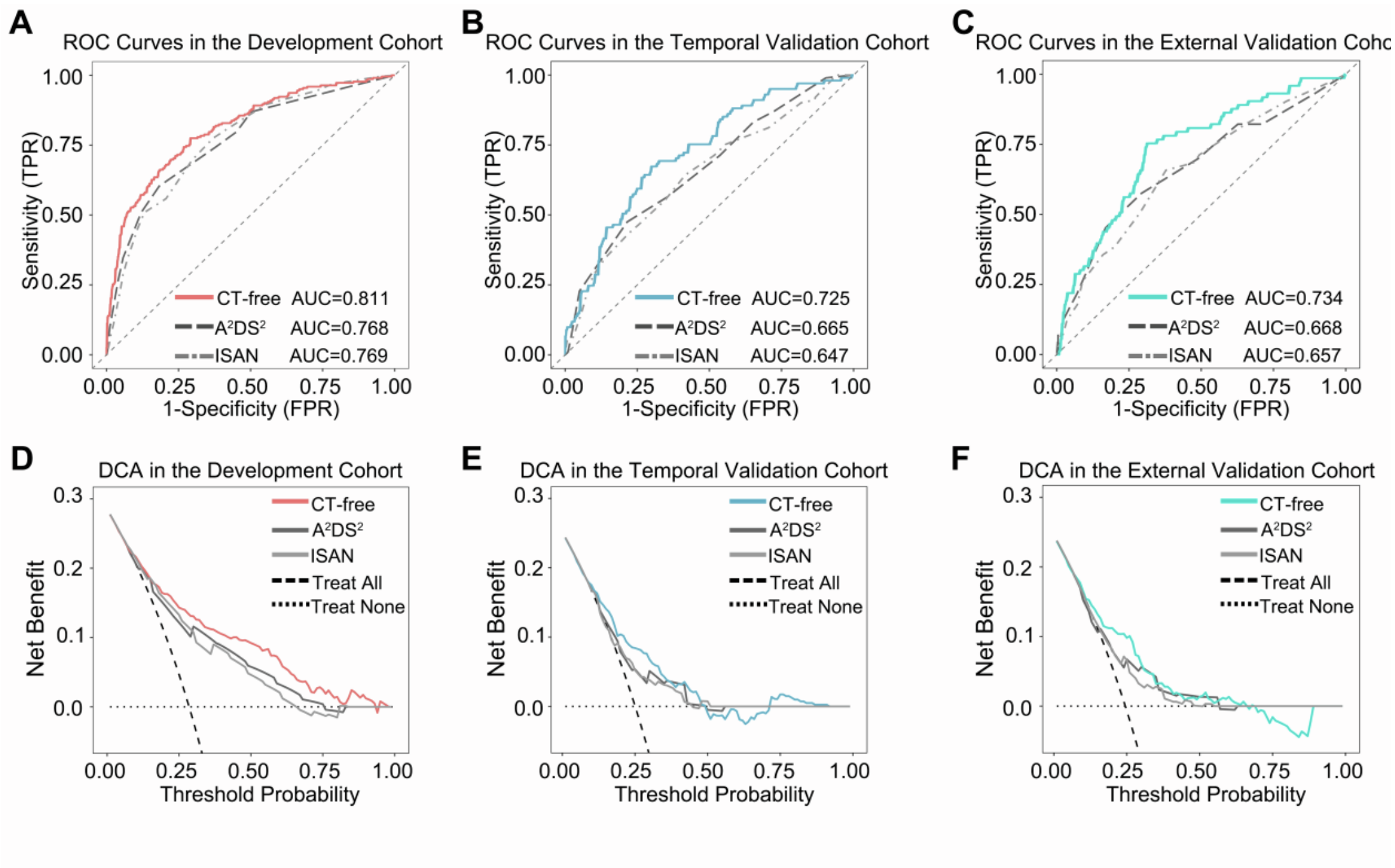

A–C: Receiver operating characteristic (ROC) curves comparing the ultra-early chest CT-free SENTRY base model with A²DS² and ISAN in the development cohort (A), temporal validation cohort (B), and external validation cohort (C). The area under the curve (AUC) is reported in each panel.

D-F: Decision curve analyses (DCA) of net benefit across threshold probabilities for the same three cohorts: development (D), temporal validation (E), and external validation (F). “Treat-all” and “treat-none” strategies are shown as reference lines.

Figure S7: Standardized Mean Differences Before and After inverse probability of treatment weighting.

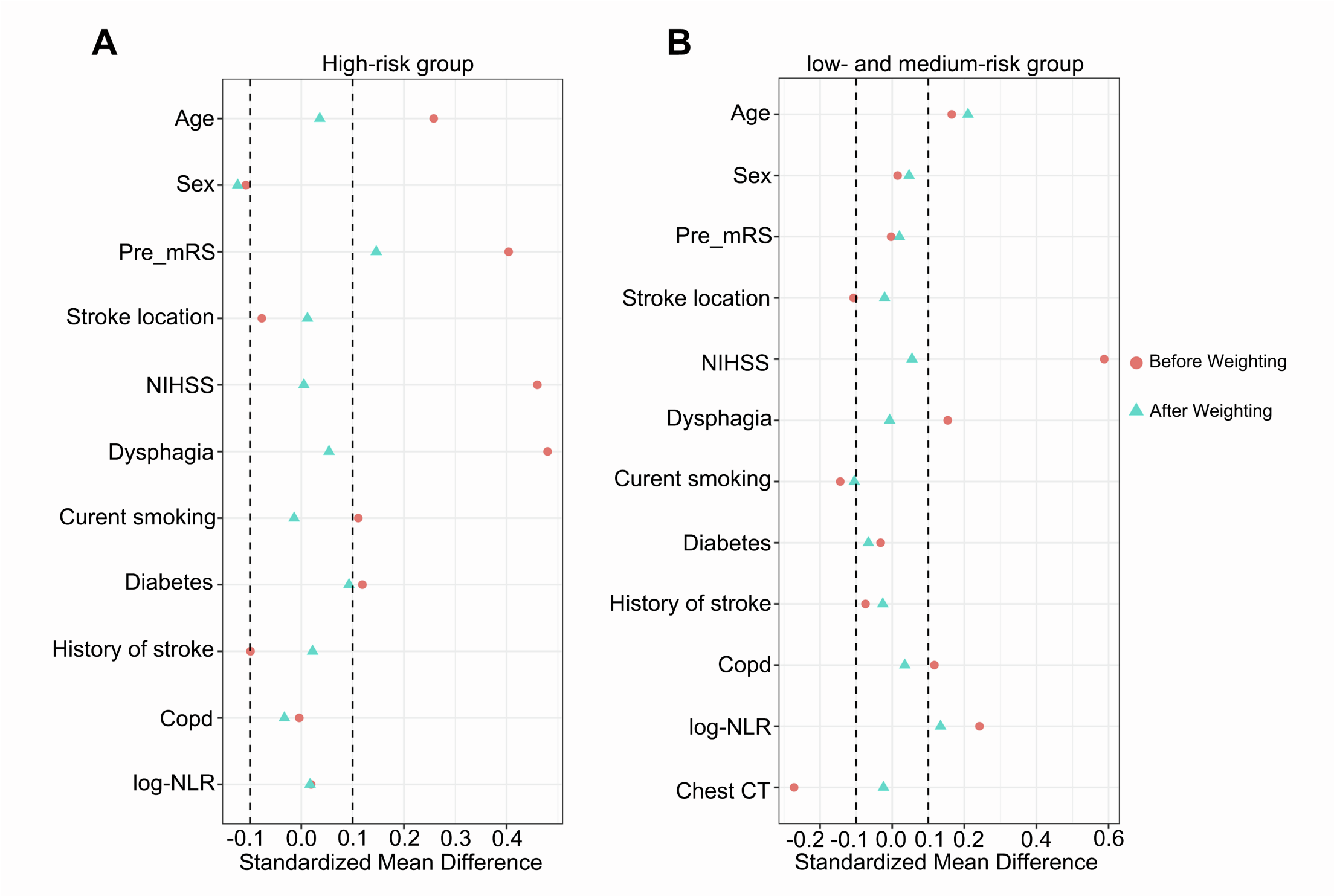

A: Standardized mean differences (SMDs) for baseline covariates in the high-risk group.

B: Standardized mean differences (SMDs) for baseline covariates in the low-and medium-risk group.

Standardized mean differences (SMDs) are shown for baseline covariates before and after inverse probability of treatment weighting. Red circles represent SMDs before weighting, and blue triangles represent SMDs after weighting.

NIHSS: National Institutes of Health Stroke Scale, log-NLR: log-transformed neutrophil-to-lymphocyte ratio (NLR).

Figure S8: Standardized Mean Differences Before and After Propensity Score Matching

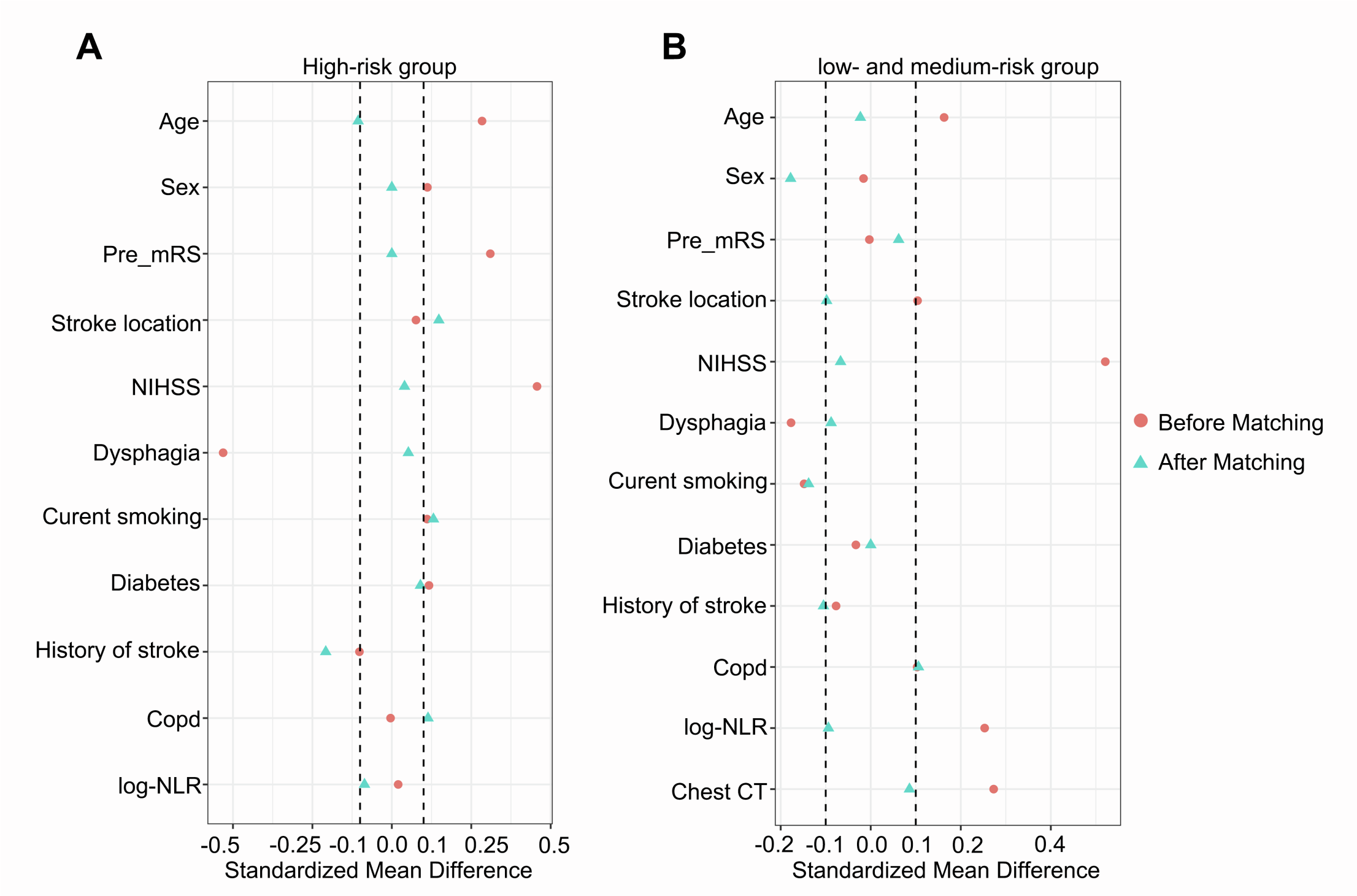

A: Standardized mean differences (SMDs) for baseline covariates in the high-risk group.

B: Standardized mean differences (SMDs) for baseline covariates in the low- and medium-risk group.

Standardized mean differences (SMDs) are shown for baseline covariates before and after propensity score matching. Red circles represent SMDs before matching, and blue triangles represent SMDs after matching.

NIHSS: National Institutes of Health Stroke Scale, log-NLR: log-transformed neutrophil-to-lymphocyte ratio (NLR).

Figure S9: Sensitivity analyses under alternative modelling assumptions.

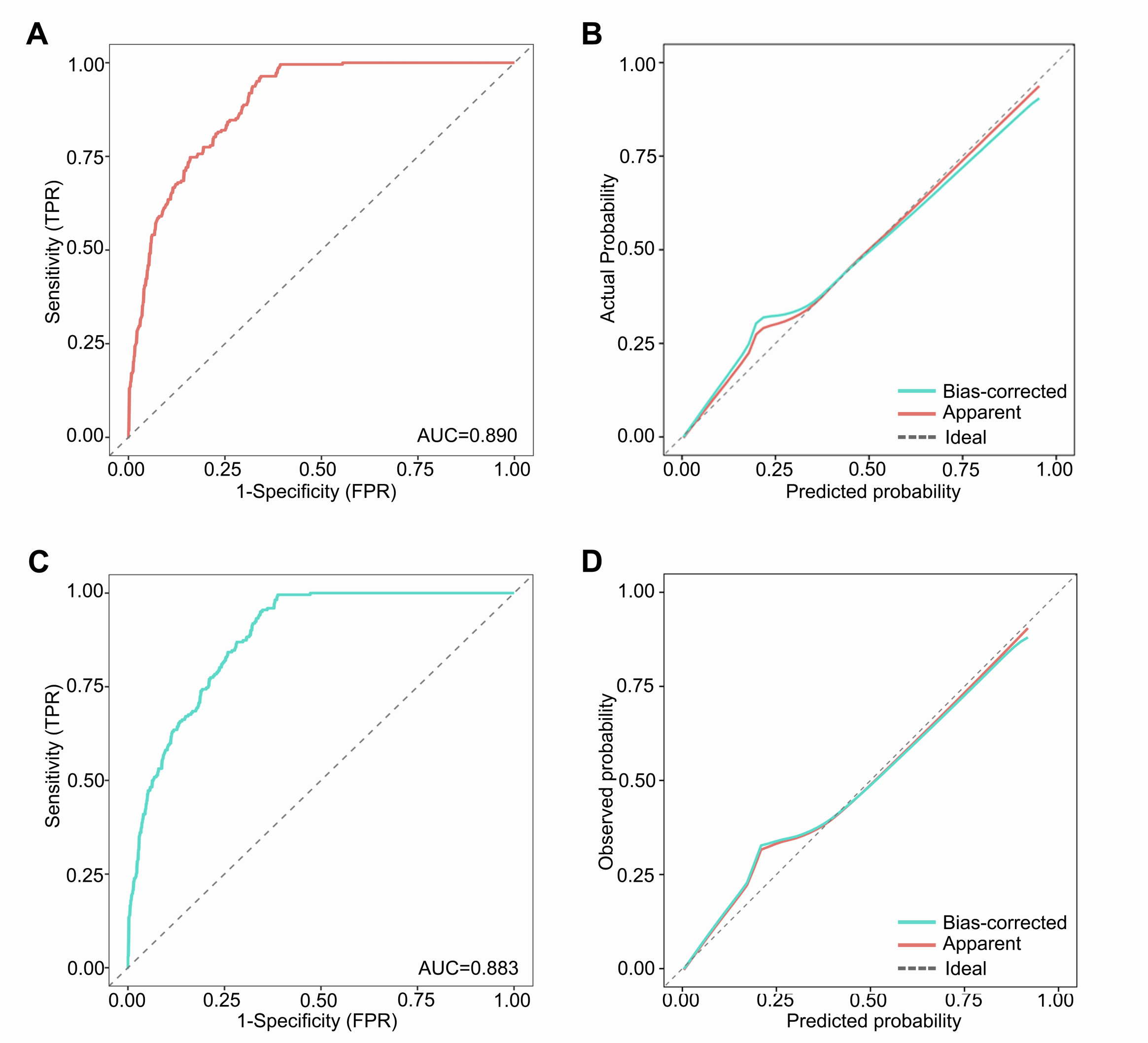

A–B: Sensitivity analysis using λ_min as the regularisation parameter.

C–D: Sensitivity analysis assuming linear effects of log-transformed NLR.

Receiver operating characteristic (ROC) curves (A, C) and calibration curves (B, D) are shown. Apparent and bootstrap bias-corrected calibration curves are presented, with the dashed line indicating ideal calibration. AUC: area under the ROC curve; NLR, neutrophil-to-lymphocyte ratio; FPR, false positive rate; TPR, true positive rate.

Figure S10: Online Tool

A:

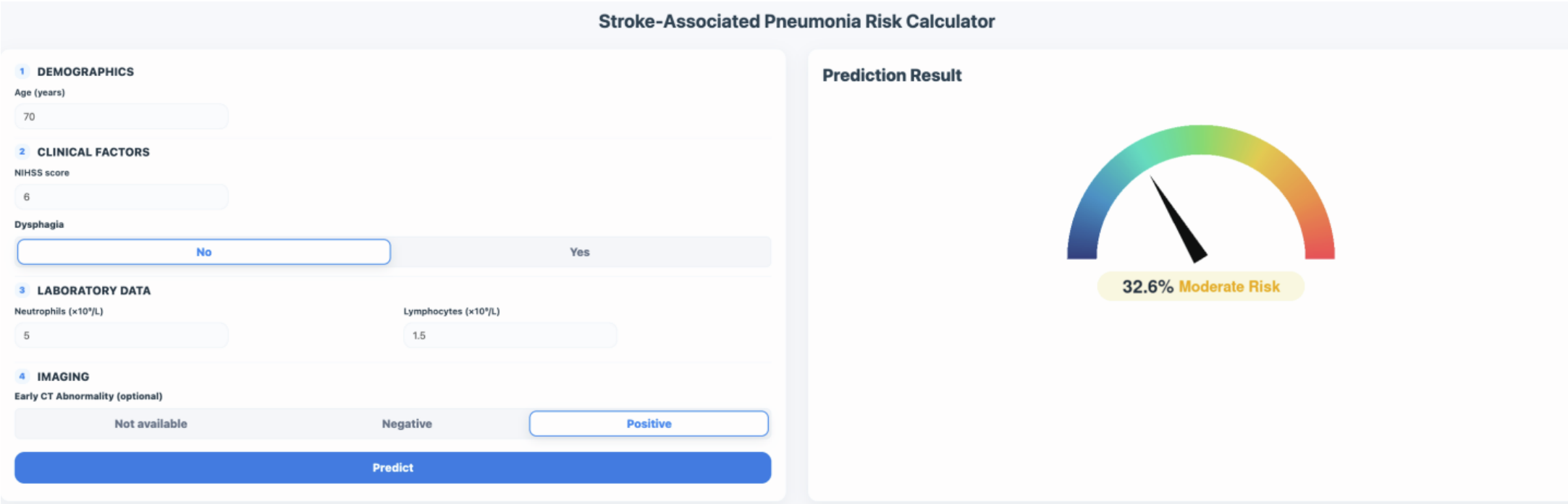

B:

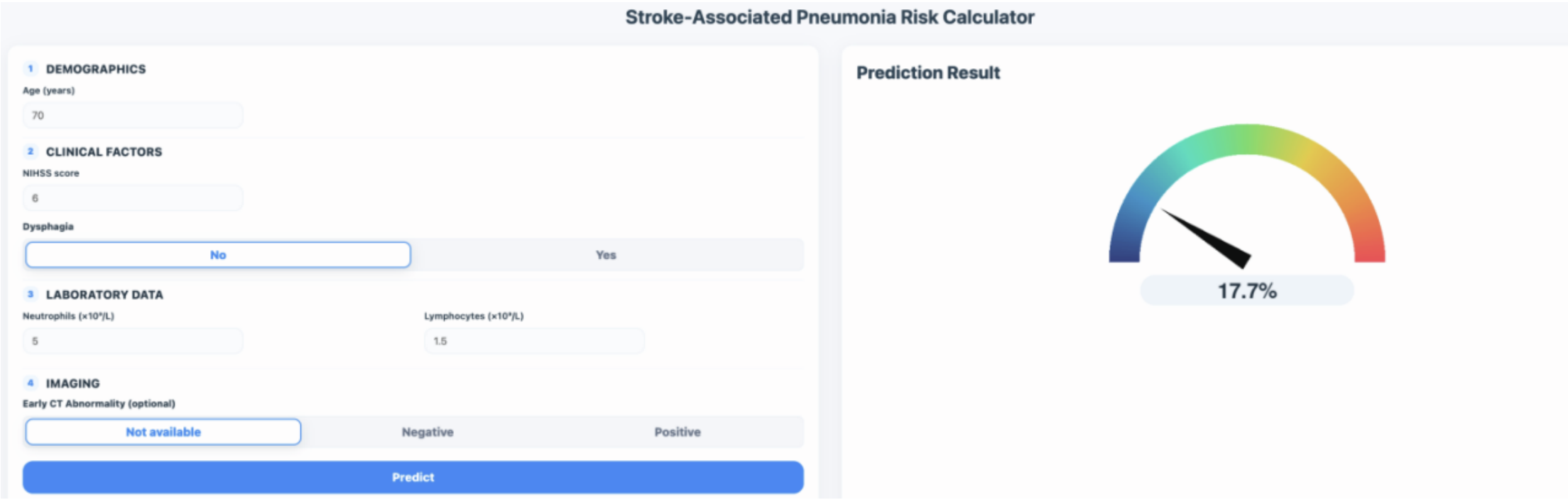

The final SAP prediction model was implemented as a web-based calculator using the Shiny framework. The calculator is designed for individuals with or without chest CT. If chest CT results are unavailable, the “not available” option can be selected for the chest CT parameter. The tool is publicly accessible at: https://sentry.shinyapps.io/SENTRY/.

A: calculator output when ultra-early chest CT abnormality is positive.

B: calculator output when chest CT results are not available.

Table S1. Leave-one-center-out internal–external validation performance

Center	Patients	Events	AUC (95% CI)	Brier score (95% CI)
1	n = 142	40	0.899 (0.849–0.949)	0.118 (0.089–0.149)
2	n = 126	36	0.884 (0.827–0.941)	0.124 (0.098–0.154)
3	n = 113	32	0.875 (0.809–0.940)	0.133 (0.101–0.166)
4	n = 129	37	0.853 (0.789–0.916)	0.141 (0.109–0.179)
5	n = 139	40	0.866 (0.807–0.925)	0.136 (0.101–0.170)
6	n = 130	37	0.878 (0.821–0.936)	0.120 (0.090–0.151)

Centers 1 to 6 are Xuanwu Hospital, Shihezi Municipal Hospital, Rongcheng County People's Hospital, The Second Hospital of Hebei Medical University, Ningjin County People's Hospital, and Suzhou Municipal Hospital.

Abbreviations: n, number of patients; AUC, area under the receiver operating characteristic curve; CI, confidence interval.

Table S2: Participating centers in three cohorts

Cohort	Center name	City and Province	Geographical location in China	Number of patients	Study period
Development (779)	Xuanwu Hospital	Beijing	North	n=142	2021.10–2022.02
	Shihezi Municipal Hospital	Shihezi, Xinjiang	North	n=126	
	Rongcheng County People's Hospital	Baoding, Hebei	North	n=113	
	The Second Hospital of Hebei Medical University	Shijiazhuang, Hebei	North	n=129	
	Ningjin County People's Hospital	Xingtai, Hebei	North	n=139	
	Suzhou Municipal Hospital of Anhui Province	Suzhou, Anhui	Central	n=130	
Temporal Validation (403)	Xuanwu Hospital	Beijing	North	n=98	2022.03–2022.06
	Shihezi Municipal Hospital	Shihezi, Xinjiang	North	n=87	
	Rongcheng County People's Hospital	Baoding, Hebei	North	n=82	
	The Second Hospital of Hebei Medical University	Shijiazhuang, Hebei	North	n=62	
	Ningjin County People's Hospital	Xingtai, Hebei	North	n=54	
	Suzhou Municipal Hospital of Anhui Province	Suzhou, Anhui	Central	n=20	
External Validation (298)	Central Hospital of Xiangtan	Xiangtan, Hunan	Southern	n=126	2022.06-2022.09
	First Affiliated Hospital of Soochow University	Soochow, Jiangsu	Southern	n=172	

Table S3: Candidate predictors

Variable domain	Variables	Type
Demographics	age（years）	Continuous
	sex (Female/Male)	Categorical
Baseline function	Pre-stroke mRS (0, 1-2, ≥3 score)	Categorical
Stroke characteristics	stroke location (Anterior circulation / Posterior circulation)	Categorical
Stroke severity	NIHSS on admission (0-40 score)	Continuous
	Dysphagia (Yes/No)	Categorical
Imaging findings	Early abnormalities in chest CT (Positive / Negative）	Categorical
Medical comorbidities	Current smoking (Yes/No)	Categorical
	Diabetes (Yes/No)	Categorical
	Hypertension (Yes/No)	Categorical
	Atrial fibrillation (Yes/No)	Categorical
	History of stroke (Yes/No)	Categorical
	COPD (Yes/No)	Categorical
Laboratory markers	Neutrophils	Continuous
	Lymphocyte	Continuous
	WBC	Continuous

Abbreviations: COPD: Chronic obstructive pulmonary disease; mRS: Modified Rankin Scale; NIHSS: National Institutes of Health Stroke Scale; WBC: white blood cell.

Table S4: Diagnostic criteria for stroke-associated pneumonia (modified CDC)

Clinical, laboratory, and image evidence	
**Radiological criteria (≥1 required)**	A new pulmonary abnormality on chest radiograph or CT:
	1.New or progressive and persistent infiltrate
	2.New consolidation
	3.Cavitation
**Systemic signs (≥1 required)**	1.Fever >38.0°C with no other cause
	2.Leukocytosis ≥12,000/mm³ or leukopenia <4,000/mm³
	3.Adults ≥70 years: acute altered mental status without another identifiable cause
**Respiratory signs (≥2 required)**	1.New onset of purulent sputum, change in sputum character, or increased respiratory secretions
	2.New or worsening cough, dyspnea, or tachypnea
	3.Rales or bronchial breath sounds on auscultation
	4.Worsening gas exchange：e.g.,$SpO_2/FiO_2 \le 240$; increased oxygen requirement; increased ventilatory support
**A diagnosis of SAP (modified CDC) requires**	1. ≥1 systemic sign, and
	2. ≥2 respiratory signs, and
	3. ≥1 new radiological abnormality.

Abbreviations: SAP: stroke-associated pneumonia; COPD: Chronic Obstructive Pulmonary Disease. ARDS: Acute Respiratory Distress Syndrome; CDC: Centers for Disease Control and Prevention.

Table S5: Comparisons of the SENTRY with A^2DS^2 and ISAN across cohorts.

Cohort	SENTRY AUC (95% CI)	A^2DS^2 AUC (95% CI)	ISAN AUC (95% CI)	p (SENTRY vs A^2DS^2)	p (SENTRY vs ISAN)
Development	0.886 (0.864 - 0.909)	0.768 (0.731-0.806)	0.769 (0.733-0.805)	<0.001	<0.001
Temporal validation	0.862 (0.821- 0.903)	0.665 (0.603-0.726)	0.647 (0.583-0.711)	<0.001	<0.001
External validation	0.874 (0.829 - 0.920)	0.668 (0.593-0.744)	0.657 (0.584-0.730)	<0.001	<0.001

Area under the receiver operating characteristic curve (AUC) with 95% confidence intervals (CIs) for the SENTRY model and two established bedside scores (A^2DS^2 and ISAN) in the development, temporal validation, and external validation cohorts. P values for between-model AUC comparisons were calculated using DeLong's test: p (SENTRY vs A^2DS^2) and p (SENTRY vs ISAN).

Abbreviations: AUC, area under the receiver operating characteristic curve; CI, confidence interval.

Table S6: Comparisons of the Base model with A^2DS^2 and ISAN across cohorts.

Cohort	Base AUC (95% CI)	A^2DS^2 AUC (95% CI)	ISAN AUC (95% CI)	p (Base vs A^2DS^2)	p (Base vs ISAN)
Development	0.811 (0.777-0.846)	0.768 (0.731-0.806)	0.769 (0.733-0.805)	0.001	<0.001
Temporal validation	0.725 (0.66-0.781)	0.665 (0.603-0.726)	0.647 (0.583-0.711)	0.017	<0.001
External validation	0.734 (0.669-0.800)	0.668 (0.593-0.744)	0.657 (0.584-0.730)	0.029	0.010

Area under the receiver operating characteristic curve (AUC) with 95% confidence intervals (CIs) for the CT-free SENTRY base model (Base) and two established bedside scores (A^2DS^2 and ISAN) in the development, temporal validation, and external validation cohorts. P values for between-model AUC comparisons were calculated using DeLong's test: p (Base vs A^2DS^2) and p (Base vs ISAN).

Abbreviations: AUC, area under the receiver operating characteristic curve; CI, confidence interval.

Table S7: Established and extensively validated SAP risk scores

Model	Year	Population	Predictors	Development	Validation	Evaluated head-to-head in our cohorts
Kwon pneumonia score[1]	2006	acute stroke	NIHSS, age, sex, mechanical ventilation, dysphagia	single center	Externally validated in multiple independent cohorts	R4
A²DS²[2]	2012	AIS	age, atrial fibrillation, dysphagia, sex, NIHSS	multicenter	Externally validated in multiple independent cohorts	yes
AIS-APS[2]	2013	AIS	age, atrial fibrillation, congestive heart failure, COPD, smoking, pre-stroke mRS, NIHSS, GCS, dysphasia, OCSP subtype, admission glucose	multicenter	Externally validated in multiple independent cohorts	R5
PANTHERIS[3]	2013	neurological intensive care unit in patients suffering from acute ischemic MCA infarction	GCS, age, white blood cell count, systolic arterial blood pressure	single center (neurological intensive care unit)	Externally validated in multiple independent cohorts	R3
ISAN[4]	2015	AIS	age, sex, NIHSS, pre-stroke mRS	multicenter	Externally validated in multiple independent cohorts	yes

Table S7 summarizes established and widely used post-stroke pneumonia risk scores that have been repeatedly evaluated in independent cohorts, focusing on scores applicable to AIS populations. Models developed exclusively in intracerebral hemorrhage populations were excluded. The table also records whether each model was evaluated head-to-head in our cohorts and, if not, prespecified reasons for non-comparison (R1–R5). Prespecified reasons for non-comparison in head-to-head analyses in our cohorts: R1, not reproducible algorithm: the publication did not provide a complete reproducible scoring equation or rules. R2, prediction window not aligned with admission-time benchmarking: The model required predictors collected after admission or outside our prespecified early prediction window (for example, 24–

48 h assessments, postoperative variables, or other time-dependent measures), preventing an admission-time head-to-head comparison. R3, selected population or care pathway: The model was developed for a restricted subgroup or specific care pathway that was not comparable to our target AIS population (for example, thrombectomy-treated AIS, AIS with atrial fibrillation, or neurocritical care cohorts), limiting fair benchmarking. R4, predictors influenced by post-admission management: The model included predictors that are influenced by in-hospital management or interventions (for example, nasogastric tube feeding or placement, mechanical ventilation, oxygen therapy), which compromises admission-time implementation and unbiased comparison. R5, predictors not available or not harmonizable in our cohorts: Key predictors were unavailable, incompletely recorded, or could not be harmonized across cohorts (for example, admission glucose, Geriatric Nutritional Risk Index, or imaging features requiring non-standard processing), preventing reliable score calculation.

Abbreviations: AIS: acute ischemic stroke; MCA: Middle Cerebral Artery; COPD: Chronic Obstructive Pulmonary Disease; mRS: Modified Rankin Scale; GCS, Glasgow Coma Scale; NIHSS: National Institutes of Health Stroke Scale; OCSP: Oxfordshire Community Stroke Project.

Table S8: Recently published SAP prediction models (published from 2016 to 2026)

Model	Year	Population	Predictors	Development	Validation	Evaluated head-to-head in our cohorts
Westendorp[5]	2018	AIS and ICH (PASS trial cohorts)	age, sex, pre-stroke mRS, COPD, NIHSS, dysphagia, type of stroke	multicenter	Internal validation (bootstrapping)	R3
Huang[6]	2019	AIS	age, admission NHISS, atrial fibrillation, nasogastric tube intervention, mechanical ventilation, leucocyte, and fibrinogen	single center	Internal validation	R4
Ge[7]	2019	AIS	demographic information, vascular risk factors, assessment of nursing levels, lab test information, medication information	single center	Internal validation	R1, R2 and R4
Li[8]	2020	AIS	age, pre-stroke mRS, NIHSS, FBG, sex, history of atrial fibrillation	single center	Internal validation	R1
Zheng[9]	2021	Stroke	diabetes, consciousness, dysphagia, invasive procedure, C-reactive protein, NLR, SIL-2R	single center	Internal validation and external validation	R4
Song[10]	2022	AIS patients with Type 2 diabetes mellitus	age, atrial fibrillation, dysphagia, sex, NIHSS, OCSP, GNRI, Hemoglobin, FBG	single center	Internal validation	R3
Yu[11]	2022	hyperacute cerebral infarction	age, atrial fibrillation, dysphagia, sex, NIHSS, previous stroke, consciousness, brain atrophy, core infarct volume (per 50 ml)	single center	Internal validation (bootstrap)	R4 and R5
Zhao[12]	2022	AIS	DWI-ASPECTS	single center	none	R5
Li[13]	2022	AIS	age, homocysteine, INR, COPD, dysphagia, walking ability within 48 h of admission	single center	none	R2 (walking ability within 48 h of admission)
Lee[14]	2023	AIS	age, NIHSS, nasogastric tube feeding, C-reactive protein	single center	none	R4 (nasogastric tube feeding)
Wang[15]	2023	AIS	age, NIHSS, atrial fibrillation, dysphagia, PLR, and GNRI	single center	Internal validation	R5 (GNRI)

Zhang[16]	2023	patients with LAA type AIS	age, NLR, NIHSS, volume-viscosity swallow test	single center	external validation	R2 and R3
Luo[17]	2024	AIS	triglycerides, NLR, dysphagia, NIHSS, internal carotid artery stenosis, radiomics score	single center	Internal validation	R2
Wang[18]	2024	AIS patients undergoing thrombectomy	postoperative LMR, NIHSS score ≥ 16, dysphagia	single center	Internal validation	R3
Zheng[19]	2024	AIS patients undergoing thrombectomy	age, inflammatory response index	single center	none	R3
Nelde[20]	2024	nontraumatic hemorrhagic and AIS	clinical and laboratory parameters along with heart rate, heart rate variability, and blood pressure values	single center	Internal validation	R2 and R5
Liu[21]	2025	AIS	age, risk of pressure injury, NIHSS, C-reactive protein	single center	Internal validation	R2 (Clinical data collected within 48 h of admission)
Su[22]	2025	AIS with atrial fibrillation	coronary heart disease, hypertension, consciousness disorder, cognitive impairment, limb movement disorders, dysphagia, nasal feeding, oxygen intake	regional multicenter	Internal validation	R3 and R4
Xie[23]	2025	AIS	MLR, immune-inflammation index, NIHSS, age, PLR	single center	Internal validation	R1

Table S8 summarizes recently published prediction models for post-stroke pneumonia from 2016 to 2026 identified from a PubMed search. Models developed exclusively in intracerebral hemorrhage populations were excluded. The table also records whether each model was evaluated head-to-head in our cohorts and, if not, prespecified reasons for non-comparison (R1–R5). Prespecified reasons for non-comparison in head-to-head analyses in our cohorts: R1, not reproducible algorithm: the publication did not provide a complete reproducible scoring equation or rules. R2, prediction window not aligned with admission-time benchmarking: The model required predictors collected after admission or outside our prespecified early prediction window (for example, 24–48 h assessments, postoperative variables, or other time-dependent measures), preventing an admission-time head-to-head comparison. R3, selected population or care pathway: The model was developed for a restricted subgroup or specific care pathway that was not comparable to our target AIS population (for example, thrombectomy-treated AIS, AIS with atrial fibrillation, or neurocritical care cohorts), limiting fair benchmarking. R4, predictors influenced by post-admission management: The model included predictors that are influenced by in-hospital management or interventions (for example, nasogastric tube feeding

or placement, mechanical ventilation, oxygen therapy), which compromises admission-time implementation and unbiased comparison. R5, predictors not available or not harmonizable in our cohorts: Key predictors were unavailable, incompletely recorded, or could not be harmonized across cohorts (for example, admission glucose, Geriatric Nutritional Risk Index, or imaging features requiring non-standard processing), preventing reliable score calculation.
Abbreviations: AIS: acute ischemic stroke; COPD: Chronic Obstructive Pulmonary Disease; mRS: Modified Rankin Scale; GCS, Glasgow Coma Scale; NIHSS: National Institutes of Health Stroke Scale; OCSP: Oxfordshire Community Stroke Project. LAA: Large artery atherosclerosis; FBG: fast blood glucose; GNRI: Geriatric Nutritional Risk Index; INR: International normalized ratio; PLR: platelet-to-lymphocyte ratio; LMR: lymphocyte-to-monocyte ratio; MLR: Monocyte-to-lymphocyte ratio; ASPECTS: Alberta Stroke Program Early CT Score.

References

1. Kwon, H.M., Jeong, S.W., Lee, S.H., and Yoon, B.W. (2006). The pneumonia score: a simple grading scale for prediction of pneumonia after acute stroke. Am J Infect Control *34*, 64–68. 10.1016/j.ajic.2005.06.011.
2. Ji, R., Shen, H., Pan, Y., Wang, P., Liu, G., Wang, Y., Li, H., Wang, Y., and China Natl Stroke, R. (2013). Novel Risk Score to Predict Pneumonia After Acute Ischemic Stroke. Stroke *44*, 1303–+. 10.1161/strokeaha.111.000598.
3. Harms, H., Grittner, U., Dröge, H., and Meisel, A. (2013). Predicting post-stroke pneumonia: the PANTHERIS score. Acta Neurol Scand *128*, 178–184. 10.1111/ane.12095.
4. Smith, C.J., Bray, B.D., Hoffman, A., Meisel, A., Heuschmann, P.U., Wolfe, C.D., Tyrrell, P.J., and Rudd, A.G. (2015). Can a novel clinical risk score improve pneumonia prediction in acute stroke care? A UK multicenter cohort study. J Am Heart Assoc *4*, e001307. 10.1161/jaha.114.001307.
5. Westendorp, W.F., Vermeij, J.D., Hilkens, N.A., Brouwer, M.C., Algra, A., van der Worp, H.B., Dippel, D.W., van de Beek, D., and Nederkoorn, P.J. (2018). Development and internal validation of a prediction rule for post-stroke infection and post-stroke pneumonia in acute stroke patients. Eur Stroke J *3*, 136–144. 10.1177/2396987318764519.
6. Huang, G.Q., Lin, Y.T., Wu, Y.M., Cheng, Q.Q., Cheng, H.R., and Wang, Z. (2019). Individualized Prediction Of Stroke-Associated Pneumonia For Patients With Acute Ischemic Stroke. Clin Interv Aging *14*, 1951–1962. 10.2147/cia.S225039.
7. Ge, Y., Wang, Q., Wang, L., Wu, H., Peng, C., Wang, J., Xu, Y., Xiong, G., Zhang, Y., and Yi, Y. (2019). Predicting post-stroke pneumonia using deep neural network approaches. Int J Med Inform *132*, 103986. 10.1016/j.ijmedinf.2019.103986.
8. Li, X., Wu, M., Sun, C., Zhao, Z., Wang, F., Zheng, X., Ge, W., Zhou, J., and Zou, J. (2020). Using machine learning to predict stroke-associated pneumonia in Chinese acute ischaemic stroke patients. Eur J Neurol *27*, 1656–1663. 10.1111/ene.14295.
9. Zheng, L., Wen, L., Lei, W., and Ning, Z. (2021). Added value of systemic inflammation markers in predicting pulmonary infection in stroke patients: A retrospective study by machine learning analysis. Medicine (Baltimore) *100*, e28439. 10.1097/md.0000000000028439.
10. Song, X., He, Y., Bai, J., and Zhang, J. (2022). A nomogram based on nutritional status and A(2)DS(2) score for predicting stroke-associated pneumonia in acute ischemic stroke patients with type 2 diabetes mellitus: A retrospective study. Front Nutr *9*, 1009041. 10.3389/fnut.2022.1009041.
11. Yu, Y., Xia, T., Tan, Z., Xia, H., He, S., Sun, H., Wang, X., Song, H., and Chen, W. (2022). A(2)DS(2) Score Combined With Clinical and Neuroimaging Factors Better Predicts Stroke-Associated Pneumonia in Hyperacute Cerebral Infarction. Front Neurol *13*, 800614. 10.3389/fneur.2022.800614.
12. Zhao, D., Zhu, J., Cai, Q., Zeng, F., Fu, X., and Hu, K. (2022). The value of diffusion weighted imaging-alberta stroke program early CT score in predicting stroke-associated pneumonia in patients with acute cerebral infarction: a retrospective study. PeerJ *10*, e12789. 10.7717/peerj.12789.

13. Li, Y.M., Zhao, L., Liu, Y.G., Lu, Y., Yao, J.Z., Li, C.J., Lu, W., and Xu, J.H. (2022). Novel Predictors of Stroke-Associated Pneumonia: A Single Center Analysis. Front Neurol *13*, 857420. 10.3389/fneur.2022.857420.
14. Lee, Y.J., and Jang, H.J. (2023). A Simple Nomogram for Predicting Stroke-Associated Pneumonia in Patients with Acute Ischemic Stroke. Healthcare (Basel) *11*. 10.3390/healthcare11233015.
15. Wang, C., Jiang, X., Wu, D., Ge, M., and Deng, L. (2023). GNRI, PLR and Stroke-Associated Pneumonia: From Association to Development of a Web-Based Dynamic Nomogram. Clin Interv Aging *18*, 1893–1904. 10.2147/cia.S433388.
16. Zhang, W.B., Tang, T.C., Zhang, A.K., Zhang, Z.Y., Hu, Q.S., Shen, Z.P., and Chen, Z.L. (2023). A Clinical Prediction Model Based on Post Large Artery Atherosclerosis Infarction Pneumonia. Neurologist *28*, 19–24. 10.1097/nrl.0000000000000434.
17. Luo, H., Li, J., Chen, Y., Wu, B., Liu, J., Han, M., Wu, Y., Jia, W., Yu, P., Cheng, R., et al. (2024). Feasibility of a clinical-radiomics combined model to predict the occurrence of stroke-associated pneumonia. BMC Neurol *24*, 45. 10.1186/s12883-024-03532-3.
18. Wang, J., Yang, C., Zhang, R., Hu, W., Yang, P., Jiang, Y., Hong, W., Shan, R., and Jiang, Y. (2024). Development and validation of a predictive model for stroke associated pneumonia in patients after thrombectomy for acute ischemic stroke. Front Med (Lausanne) *11*, 1370986. 10.3389/fmed.2024.1370986.
19. Zheng, F., Gao, W., Xiao, Y., Guo, X., Xiong, Y., Chen, C., Zheng, H., Pan, Z., Wang, L., Zheng, S., et al. (2024). Systemic inflammatory response index as a predictor of stroke-associated pneumonia in patients with acute ischemic stroke treated by thrombectomy: a retrospective study. BMC Neurol *24*, 287. 10.1186/s12883-024-03783-0.
20. Nelde, A., Krumm, L., Arafat, S., Hotter, B., Nolte, C.H., Scheitz, J.F., Klammer, M.G., Krämer, M., Scheib, F., Endres, M., et al. (2024). Machine learning using multimodal and autonomic nervous system parameters predicts clinically apparent stroke-associated pneumonia in a development and testing study. J Neurol *271*, 899–908. 10.1007/s00415-023-12031-3.
21. Liu, X., Mo, J., Liu, Z., Ge, Y., Luo, T., and Kuang, J. (2025). Enhancing stroke-associated pneumonia prediction in ischemic stroke: An interpretable Bayesian network approach. Digit Health *11*, 20552076251333568. 10.1177/20552076251333568.
22. Su, T., Zhang, P., Zhang, B., Liu, Z., Xie, Z., Li, X., Ma, J., and Xin, T. (2025). Risk prediction of stroke-associated pneumonia in acute ischemic stroke with atrial fibrillation using machine learning models. Front Artif Intell *8*, 1595101. 10.3389/frai.2025.1595101.
23. Xie, M., Liu, Z., Dai, F., Cao, Z., and Wang, X. (2025). Predicting Stroke-Associated Pneumonia in Acute Ischemic Stroke: A Machine Learning Model Development and Validation Study with CBC-Derived Inflammatory Indices. Int J Gen Med *18*, 3117–3128. 10.2147/ijgm.S524450.
